# Use of the first-acquired language modulates pupil size in the processing of island constraint violations

**DOI:** 10.3389/fpsyg.2023.1180989

**Published:** 2023-07-14

**Authors:** Gita Martohardjono, Michael A. Johns, Pamela Franciotti, Daniela Castillo, Ilaria Porru, Cass Lowry

**Affiliations:** ^1^Department of Linguistics and Communication Disorders, Queens College, New York, NY, United States; ^2^Second Language Acquisition Laboratory, Linguistics Program, The Graduate Center of the City University of New York, New York, NY, United States; ^3^Institute for Systems Research, University of Maryland, College Park, MD, United States

**Keywords:** pupillometry, heritage speakers, late bilinguals, current use, historical use, group vs. individual analyses

## Abstract

**Introduction:**

Traditional studies of the population called “heritage speakers” (HS) have treated this group as distinct from other bilingual populations, e.g., simultaneous or late bilinguals (LB), focusing on group differences in the competencies of the first-acquired language or “heritage language”. While several explanations have been proposed for such differences (e.g., incomplete acquisition, attrition, differential processing mechanisms), few have taken into consideration the individual variation that must occur, due to the fluctuation of factors such as exposure and use that characterize all bilinguals. In addition, few studies have used implicit measures, e.g., psychophysiological methods (ERPs; Eye-tracking), that can circumvent confounding variables such as resorting to conscious metalinguistic knowledge.

**Methodology:**

This study uses pupillometry, a method that has only recently been used in psycholinguistic studies of bilingualism, to investigate pupillary responses to three syntactic island constructions in two groups of Spanish/English bilinguals: heritage speakers and late bilinguals. Data were analyzed using generalized additive mixed effects models (GAMMs) and two models were created and compared to one another: one with group (LB/HS) and the other with groups collapsed and current and historical use of Spanish as continuous variables.

**Results:**

Results show that group-based models generally yield conflicting results while models collapsing groups and having usage as a predictor yield consistent ones. In particular, current use predicts sensitivity to L1 ungrammaticality across both HS and LB populations. We conclude that individual variation, as measured by use, is a critical factor tha must be taken into account in the description of the language competencies and processing of heritage and late bilinguals alike.

## 1. Introduction

Research on heritage speakers (HS) over the past 20 years has claimed that these childhood bilinguals whose home language is a societal minority language, differ qualitatively in the competence of their first-acquired language^1^ (henceforth L1) when compared to other bilinguals (Benmamoun et al., 2013a; Montrul, 2016b; Polinsky, 2018). Specifically, HS are said to diverge in their L1 production (e.g., Fenyvesi, 2005), comprehension (e.g., Polinsky, 2006), lexical diversity (e.g., Hulsen, 2000), and grammatical intuition (e.g., Montrul and Bowles, 2009). This observed variation has led some researchers to the hypothesis that HS are a distinct type of bilingual due to the early age of initial exposure to the L2, although other factors, such as proficiency and attrition have also been suggested (Polinsky, 2016; Montrul, 2022).

While the majority of the HS literature documents behavioral outcomes in the L1, much less is known about heritage language processing from a psycholinguistic perspective, though initial investigations provide evidence that HS processing has both similarities and differences with the processing patterns of other bilingual populations who share their L1 (Madsen, 2018; Martohardjono et al., 2021). The goal of our study is to further the investigation into HS processing by investigating the role of *relative language use* in Spanish/English bilinguals and how it may affect processing of the first-acquired language, Spanish. We take as our starting point the widely stated observation that the bilingual experience is largely determined by the relative interaction with the two languages, and that this interaction can vary greatly from one speaker to the next (Grosjean and Li, 2013), suggesting that individual variation plays a critical role (see also Rothman et al., 2023). We argue that while the categorization of bilingual speakers into distinct types, such as childhood/early/heritage on the one hand, and adult/late on the other, may be intuitively appealing, especially when viewed from the perspective of critical or sensitive periods of language acquisition, it critically ignores the fact that the bilingual experience varies systematically along many dimensions other than age, such as linguistic environment, exposure, input and use. These factors have only recently been included as variables in experimental studies of bilingualism (see section 2.2.2) and our study aims to contribute to this line of inquiry.

More so than any other bilingual “type,” HS bilinguals have primarily been described in the literature as being dominant in the later-acquired, societal majority language (henceforth L2, e.g., Benmamoun et al., 2013b). But language dominance is itself a complex concept determined by a number of factors, such as age of onset (AoO), proficiency, lifetime exposure, use and contexts of use (Montrul, 2016a). Turning specifically to use factors, the variable of focus in our study, we note that dominance in one language, more often than not, entails diminished use of the other, which in turn may affect its processing (Putnam and Sánchez, 2013). The question that arises then is, does relative use (of the L1 and the L2) affect processing of the L1, and if so, how? Moreover, while L2 dominance may characterize many or most heritage speakers, use of the heritage language (HL) can vary widely. For some, use of the HL is restricted to a limited number of domains, such as family and in particular, elders, thus also limiting the scope of its use. Others, however, are raised and continue to live in a vibrant bilingual community where the HL, in spite of being a societal minority language, is used daily and in a variety of contexts. For these HS, use of the HL may remain high. Therefore, there is likely to be variability in HL use across HS populations, something that has largely been ignored in the HL literature. But HS are not the only bilingual population susceptible to variable use of the L1, as has been amply attested in L1 attrition studies (e.g., Schmid, 2011). Late bilinguals (LB), i.e., those whose acquisition and active use of the L2 occurs only later in life for a variety of reasons, such as university study, work, migration, etc. may also experience variable L1 use. A first step then, is to investigate to what degree relative use of the two languages affects processing of L1 in two groups of bilinguals, HS and LB, who are otherwise only distinguished by age of onset of the L2. If it turns out that use factors affect the two groups in similar ways, the classification of HS as a distinct bilingual “type” becomes less compelling as it may simply be the case that increased use of the L2 has affected processing of the L1 while keeping competence relatively intact. The main innovation we bring to the field of heritage speaker studies, then, is the inclusion of *relative use* as a potential predictor of how the L1 is processed. A second innovation is the application of a methodology that has only recently been introduced in the study of bilingualism and indeed, language in general, namely pupillometry.

The current study is part of a larger project investigating HS and LB who are fluent in both their L1, Spanish, and their L2, English. The HS recruited for this project were either born or had arrived in the US before age 5 and had Spanish as their home and community language. They were schooled in the L2 English starting around age 4 (pre-Kindergarten) and while some became dominant in the L2, they continued to maintain and use their L1. This group was therefore classified as having an early onset of bilingualism. The participants grouped as LB, on the other hand, were born in a Spanish-speaking country, were schooled in Spanish and immigrated to the US in adulthood. While some had limited classroom instruction in English as part of their high school curriculum, this did not occur before age 12. They were fully immersed in English only upon arrival to the US, which for most occurred in their 20s. While everyone in this group had become fluent in the L2 English by the time of testing, they had a late onset of bilingualism, both because they were first exposed to the L2 after age 12 and because they did not have active use of the L2 until adulthood.

The overall purpose of the project is to investigate differences and similarities between HS and LB in the processing of complex sentences (relative clauses and wh-questions) in L1 Spanish. Both implicit (Visual World Paradigm (VWP), EEG, pupillometry) and explicit measures (response accuracy, metalinguistic/acceptability judgments) were taken and compared. Individual-level characteristics were collected in an extensive questionnaire. In the study reported here, we present data from pupillary responses to grammatical and ungrammatical wh-questions involving island constraints (see section 2.3.2). Previous analyses of ERP data on similar structures have been reported in Phillips et al. (2021), and of pupillometry data in Martohardjono et al. (2021). Relevant results from these studies will be discussed in comparison to the results of the present study.

We begin with the characterization of heritage language speakers typically adopted in the literature, as a distinct bilingual “type.” This is followed by a summary of studies that investigate the role of relative use and exposure as determining factors in bilingualism. We then motivate the present study and describe two previous studies we conducted on the processing of wh-questions. This section also includes a description of the use of pupillometry in language studies. We then lay out the present study, including analyses and results. We conclude with a discussion of the results and general conclusions.

## 2. Background and rationale

### 2.1. Heritage speakers as a cognitively distinct bilingual type

The group commonly known as Heritage Speakers consists of children of immigrants in a particular situation of first language acquisition, involving majority vs. minority language settings. As such, they are raised in the home language, which is the societal minority language, until they reach school age, when they begin education in the societal majority language. Many, though not all, heritage speakers become dominant in that language. Nonetheless, we note that heritage speakers often retain fluency in the home language, depending on their particular linguistic environment—for example if they live in a community where maintenance of the minority language is prevalent, leading to sustained use. This is often the case in Hispanic communities in the US (Otheguy and Zentella, 2011).

Early studies described HS (Benmamoun et al., 2013a) as being qualitatively distinct in their bilinguality^2^ from LB, who are thought to have a more uniform and continuous experience of their first language, are schooled in that language, and acquire the other language only later in life. For example, it was argued that heritage speakers are distinct from child first language learners, and that the particular conditions under which they learn the home language often leads to interrupted, “incomplete acquisition” of that language (see for example Montrul, 2008, 2022). In recent years, this deficit-framing of heritage speakers’ acquisition of their home language has faded in the literature, being replaced with more neutral terms such as “differential acquisition” (Kupisch and Rothman, 2018), and “divergent attainment” (Polinsky and Scontras, 2020). Furthermore, the notion of incompleteness has been challenged by some (e.g., Bayram et al., 2019; Higby et al., 2023) and several studies have reported full acquisition of various aspects of the heritage language grammar (e.g., Guijarro-Fuentes and Schmitz, 2015; Schmitz et al., 2016; Schmitz and Scherger, 2019).

While all bilinguals are susceptible to attrition and cross-linguistic influence—two phenomena common in cases of language contact—HS are in general thought to be even more so (but for counter-examples, see Chang et al., 2011^3^) since in the process of becoming dominant in the L2, the mental representation and processing of the L1 can weaken (e.g., Gallo et al., 2021). But the claim that HS bilinguals are *as a group* distinct from other bilingual types implies a significant degree of homogeneity, presumably of a cognitive nature, due to early exposure to the L2. While some argue that this cognitive difference is representational (Polinsky, 2016), others argue that it is primarily located in the processing mechanism (Putnam and Sánchez, 2013; Hopp and Putnam, 2015). Our study does not seek to address that debate directly. It is indeed possible that restructuring of the L1 grammar occurs in some heritage speakers, and that this is likely due to the demands of having to process the two languages continuously. However, restructuring is by no means a phenomenon that is unique to heritage speakers. Competing demands are faced by all bilinguals, including those who acquire the L2 late in life but become fluent in it. As a result, restructuring of the L1 grammar may occur, i.e., attrition. Here we focus instead on *processing* of the L1 and contrast two factors that could arguably affect it. The first is Age of L2 immersion (e.g., Kałamała et al., 2022), which we use as the criterial factor separating HS and LB, early for HS (usually around 6) later for LB (usually after a purported critical period). This comparison will involve a group analysis. The second factor is relative use of L1/L2 which will involve a continuous variable analysis collapsing HS and LB. As there is ample evidence from neuro- and psycholinguistic studies that proficiency in a language modulates its processing (McLaughlin et al., 2010; Morgan-Short et al., 2012a,b; Alemán Bañón et al., 2018) we keep proficiency constant across all participants, including only those who have a self-rated score of 4/5 or higher in the L1.

### 2.2. Language use as a variable in bilingual studies

#### 2.2.1. Neurolinguistic studies

Although research into relative language use in bilinguals is fairly recent, it has yielded interesting results in a variety of domains. For example, in a number of neurolinguistic studies, Pliatsikas and colleagues have shown that use has structural repercussions. Pliatsikas et al. (2020) proposed a three-stage model for language acquisition and use. When participants are first exposed to a second language, gray matter volume in vocabulary-learning and language-control regions increases (stage 1) but proliferation of these regions fades with L2 experience. During stage 2, language-controlling subcortical and cerebellar adjustments emerge (Abutalebi and Green, 2016) but these adaptations should also fade, possibly resulting in pruning processes and white matter adaptations, indicating less frontal lobe engagement and, consequently, more automation (stage 3).

DeLuca et al. (2019) investigated the effect of exposure and use in bilinguals with a wide range of age of second language acquisition (AoL2A; 0–22 yrs) living in an L2 English majority environment. Two models were compared: the first model included duration (L2 AoA and Length of L2 immersion) and degree/extent of bilingual language use (i.e., L2 exposure and use in the home and other social contexts) as predicting variables. The second model investigated active use of the L2 (total number of years actively using the L2) and immersion (length of time actively using the L2 in immersion settings). Results from both models predicted adaptations to subcortical structure. Specifically, results indicated that sustained active use of the L2 induces structural changes thought to optimize efficacy in L2 processing and production.

The effect of language use on brain structures is also evident in late sequential bilinguals. In two studies comparing highly proficient bilinguals with either high or limited immersion against two groups of monolinguals, Pliatsikas et al. (2017) found subcortical expansion changes in the highly immersed bilingual group compared to the monolingual group, whereas the non-immersion group showed insubstantial changes in comparison to the monolingual speakers. These results suggest that amount of immersion in a bilingual environment has structural correlates in the brain.

#### 2.2.2. Behavioral and psycholinguistic studies of relative language use

A number of studies using behavioral and psycholinguistic measures have investigated whether higher language use leads to faster language processing (e.g., De Bruin et al., 2016); and whether language use interacts with proficiency regardless of age of first language exposure (De Carli et al., 2015). Other studies investigated the role of language use from a methodological perspective arguing for this factor to be included when quantifying bilingualism through language background questionnaires (e.g., Luk and Bialystok, 2013; Kałamała et al., 2022).

De Bruin et al. (2016) investigated the effect of language use in three groups of older Gaelic-English speakers whose L1 is Gaelic. They were categorized as *active* bilinguals (equal use of both languages), *inactive* bilinguals (higher use of English than Gaelic) and monolinguals (very little use of Gaelic across the lifespan). Accuracy and response times (RTs) of the three groups were compared while performing a picture-word matching task in both English and Gaelic. In the English task, they found that while all groups were highly accurate, differences emerged in terms of processing speed. When self-rated English use was treated as a continuous variable, the authors report a significant effect of current language use, namely participants who reported a higher use of English had faster RTs in the English task. In the Gaelic task, findings showed that the inactive group was less accurate than the active group of bilinguals and that the RT difference between Gaelic (the L1) and English (the L2) was larger than in the active group, suggesting that current language use plays a more significant role than early use.

De Carli et al. (2015) investigated and compared the effect of language use and age of acquisition (AoA) on the language proficiency of bilinguals. They administered a sentence recognition task to two groups of speakers: Italian-Spanish bilinguals and highly proficient Spanish and Italian L2 speakers with L1 Italian and L1 Spanish, respectively. Based on current use of each language (Italian and Spanish) across different contexts and according to their responses, participants were classified into two subgroups of users, *occasional* and *intensive* users. In the sentence recognition task, participants were presented with an Italian or Spanish sentence (i.e., “Me gustaría dar un paseo,” *I would like to take a walk*) together with two alternative translations in the other language, an incorrect one (i.e., “Mi piacerebbe dare un passaggio,” *I would like to give a ride*) and a correct one (i.e., “Mi piacerebbe fare una passeggiata”). Findings showed no effect of AoA but a significant effect of language use in both RTs and accuracy. Early bilinguals who keep using both languages intensively were faster and more accurate, as were L2 speakers who were also intensive users of both languages, with no significant differences between the two groups. This suggests that AoA had little if any effect for these groups. Intensive bilingual users were also significantly faster and more accurate in their responses when compared to occasional bilingual users who did not statistically differ from the L2 speakers.

In a study on Polish-English bilinguals living in Poland and using English on a daily basis Kałamała et al. (2022) investigated the relationship between different measures of bilingualism: Onset of Bilingualism (L2 AoA), L2 Age of Active Communication (AoAC), L2 proficiency, daily use of L2 (time spent using the L2) and patterns of language use (language entropy/diversity of language use, code mixing, code switching).^4^ More specifically, the authors aimed to establish which aspects of bilingualism best predict L2 abilities. Language use and diversity of language use were assessed through two questionnaires each asking about use in several contexts. Many findings were reported, but significant for the purposes of our study were the following: while AoA predicted self-confidence in using the L2 (earlier AoA, higher self-confidence), higher L2 use was a significant predictor of greater vocabulary knowledge; bilinguals with a more diverse language use tend to be more confident in the use of the L2 but have poorer vocabulary knowledge. Finally, frequent language switchers tended to have better vocabulary knowledge, though the effect was modulated by AoA and found only in late bilinguals. Overall, Kałamała et al.’s findings suggest that diversity of language use (language entropy) and AoA affect self-confidence in using the L2 and that diversity of language use, greater language use, and language switching practices (in late bilinguals only) have an impact on vocabulary knowledge.

The picture that emerges from the above is that the degree of interaction with a language, whether defined as use, current use, diversity in use (language entropy), exposure, or immersion, has distinct outcomes in neural structure, processing (reaction times), and proficiency (accuracy) in both early and late bilinguals. In the following section we return to the question of how this plays out in two purportedly distinct Spanish/English bilingual populations, HS and LB, focusing on processing of the L1 Spanish.

### 2.3. Preliminary experimental evidence on HS processing

There is preliminary evidence that HS process their L1 differently from both native speakers and late bilinguals, due to early exposure to and use of their L2. Auditory perception studies show that balanced early bilinguals, compared to late bilinguals, have more difficulty processing their L1 in noisy environments (Weiss and Dempsey, 2008) or discriminating phonological categories (Peltola et al., 2012). Semantic judgment tasks show that early bilinguals are slower to categorize semantically anomalous items than late bilinguals and monolinguals with the same L1 (Proverbio et al., 2007).

Our own studies suggest that HS show divergent L1 processing patterns compared to LB in both grammatical and ungrammatical sentences. In an VWP experiment, HS of L1 Spanish did not show an expected sensitivity to relative clause type (subject vs. object RC), which LB did (Madsen et al., 2019). Similarly, in pupillometric studies of relative clause processing, late bilinguals showed an expected increase of processing cost for object relative clauses (increased pupil diameter), but HS did not (Madsen, 2018). In a study using event-related potentials, HS showed a sensitivity to different relative clause types, but their pattern of ERP components differed from that of LB (Madsen, 2018). Importantly, the HS tested in these studies all had high levels of proficiency in their L1, similar to that of the LB comparison group. As already mentioned, this was intentional, as we wanted the variable of comparison to be Onset of Bilingualism (AoA of the L2), not L1 proficiency. Taken together, these results suggest that HS’ increased dominance in their L2 due to increased early exposure to their L2 has large effects in their syntactic processing of the L1 (see also Montrul, 2016a). However, we subsequently found that when predictor variables of use are included, a more nuanced picture emerges. In a series of studies comparing L1 Spanish/L2 English HS and LB groups we investigated knowledge and processing of L1 ungrammaticality through metalinguistic judgments, EEG, and pupillometry. As in our previous studies, we only included participants who were fluent in both L1 Spanish and L2 English since our critical variable was onset of bilingualism, proxied as age of arrival in the US (HS/early vs. LB/late) and importantly NOT L1 proficiency. A second reason to have fluency as a criterion is the complexity of the particular structures we tested, namely grammatical and ungrammatical wh-questions containing different types of subordinate clauses. Participants classified as LB started active use of English in adulthood while those classified as HS did so at school age. As these studies are relevant to the current one, we will describe them in some detail below.

#### 2.3.1. ERP responses to L1 (un)grammaticality

In an ERP study investigating the processing of syntactic structures that contrast in grammaticality between the L1 Spanish and the L2 English, Phillips et al. (2021) performed two analyses on the same dataset of aurally presented wh-questions in Spanish. The first analysis was based on group differences of L2AoA (LB vs. HS); the second on individual variables of language history and use across the two groups. Spanish and English show a contrast in the obligatory use of the complementizer *que/that* in questions containing embedded clauses.^5^

1)  Sarah said (that) Lindsey is going to the party.

    Who_i_ did Sarah say (*that) ____i_ is going to the party?

2)  Isabel dijo *(que) Julieta va a la fiesta.

    ¿Quién_i_ dijo Isabel *(que)____i_ va a la fiesta?

                                                                                                (examples from

                                                                                                Phillips et al., 2021)

Results showed that Spanish wh-questions without a complementizer, evoked an N400 in the LB group but not in the HS group. This suggests that HS processing of these L1 structures is influenced by the L2 English, where an N400 component would not be expected for the equivalent English sentence, supporting the claim that HS as a group hold qualitatively different representations of the L1 Spanish than LB.

The second analysis examined whether individual variables collected in an extensive questionnaire for the same participants were predictive of sensitivity to the (un)grammaticality of these sentences. Predictor variables included current use of L2 English, exposure to L2 English over time, in different settings and with different interlocutors, and L2AoA (LB or HS). Results show that N400 amplitude to ungrammatical L1 Spanish sentences decreased as English use and exposure increased, indicating that increased L2 use diminished sensitivity to ungrammaticality in the L1 Spanish. Crucially, the group variable was not predictive. That is, regardless of whether a subject had early L2AoA (was grouped as HS) or late (was grouped as LB), the amount of L2 English exposure and use influenced processing of L1 Spanish. This result aligns with previous studies using eye-tracking and showing cross-linguistic influence from the L2 on the processing of L1 relative clause attachment (Dussias and Sagarra, 2007) evidencing “permeability” of the L1 after prolonged exposure to an L2.

#### 2.3.2. Pupillometric responses to L1 violations of island constraints

The data we present in the current report are based on a previous pupillometry study which we describe here, comparing LB and HS on island constraints. In that study, we used two separate tasks, administered in separate sessions, 10 to 14 days apart: an acceptability judgment task and a pupillometry task on auditorily presented Spanish sentences varying in (un)grammaticality along a hierarchy known in the syntactic literature as “strong” and “weak” islands (Martohardjono et al., 2021). These structures have been extensively studied in the L2 acquisition literature (e.g., Belikova and White, 2009; Kush and Dahl, 2022), within a native speaker processing framework (e.g., Hofmeister et al., 2013) and within the framework of experimental syntax (Sprouse and Hornstein, 2013). Strong islands included wh-questions out of relative clauses and temporal adverbials which result in a high degree of unacceptability. Weak islands included wh-questions out of wh-islands (e.g., when/how/why) and noun complements. Samples of strong (indicated with **) and weak (indicated with *) islands as illustrated in 3) below were tested against their grammatical counterparts in auditory mode and participants were asked to judge them on a scale of 1–5 for acceptability.

3)  Strong Island

        Grammatical:

        a. ¿Qué niño comió el dulce mientras que su tía buscaba la comida?

            ‘Which child ate the candy while his aunt looked for food?’

        Strong ungrammatical:

        b. **¿Qué tía_i_ el niño comió el dulce mientras que ____i_ buscaba la comida?

            ‘Which aunt did the child eat the candy while looked for food?’

    Weak Island

        Grammatical:

        a. ¿Qué enfermera confirmó Ignacio que había llevado la medicina?

            ‘What nurse did Ignacio confirm had brought the medicine?’

        Weak ungrammatical:

          b. *¿Qué enfermera_i_ confirmo Ignacio por qué ____i_ habia llevado la medicina?

             What nurse did Ignacio confirm why had brought the medicine?’

Results of the acceptability judgment task showed almost parallel behavior for LB and HS, with significantly higher rejection rates for all ungrammatical structures in both weak and strong conditions, when compared to their grammatical counterparts. This was interpreted as the two groups sharing metalinguistic intuitions about these sentences.

The pupillometry results were more complex, with group means for LB and HS showing partly different pupil dilation patterns. For wh-islands, a weak condition, neither LB nor HS showed the expected increase in pupil dilation for ungrammatical sentences. In fact, both groups showed the reverse pattern, with larger dilation for grammatical than ungrammatical sentences. LB and HS showed slightly different patterns for the other weak constraint, noun complements, although neither in the expected direction. LB showed no significant differences between grammatical and ungrammatical conditions, while HS showed again the reverse pattern, with grammatical sentences eliciting larger pupil dilation than ungrammatical ones, an unexpected result. For the strong constraints, LB and HS converged only in the relative clause condition, with both groups showing a significant increase in pupil dilation for ungrammatical sentences compared to grammatical sentences. In the temporal adverbial type, LB showed the expected pattern, while HS showed no significant differences between grammatical and ungrammatical conditions.

The conclusion we drew from the group analysis of the judgment and pupillometry tasks was that (1) in bilingual populations, processing patterns do not always align with metalinguistic patterns, (2) that the greater between-group differences in processing for LB vs. HS may be reflective of age of L2 acquisition differences, although this was not seen in acceptability judgments, and (3) that the unexpected dilation patterns may be related to the (un)interpretability, rather than the (un)grammaticality of a sentence. Together, our two previous studies indicate that while explicit, metalinguistic knowledge (as measured by judgments) largely coincide across fluent Spanish/English bilinguals, regardless of onset of bilingualism (i.e., HS/LB), processing patterns may in fact diverge across the two groups, lending credence to the claim that the two groups can indeed be considered distinct at some level. However, when use variables are factored in, as they were in the ERP study, these turn out to have an influence on syntactic processing that overrides that of group categorization.

The present study is a follow-up to the ERP and pupillometry studies we just described. In particular, given that metalinguistic judgments of island violations did not differ between HS and LB, but group analyses of the pupillometric data gave inconsistent and even puzzling results; and given further that in the ERP study we found usage factors significantly modulating L1 processing of ungrammaticality (N400 amplitude) in a structure of L1/L2 contrast (obligatory vs. optional complementizer), we wanted to see (1) whether usage factors might also play a role in determining sensitivity to violations that hold in both languages and (2) whether a model using only usage factors as terms might shed light on the unexpected and puzzling (group) results found in the previous study. Before delving into the details of the present study in section 3, we give a brief description of how pupillometry has been applied in language studies, since it is a fairly recent addition to the methodologies used in the field (e.g., Scherger et al., 2021).

### 2.4. Pupillometry in linguistic research

Pupillometry is known to be an implicit measurement that allows to track cognitive processes online without relying on explicit responses. Pupil dilation has long been associated with higher cognitive load when completing a task, i.e., the higher the effort the greater the change in pupil dilation. This has been well-attested for roughly half a century in several pioneering studies using this methodology in non-linguistic research (e.g., Hess and Polt, 1964; Kahneman and Beatty, 1966; Schmidtke, 2018 for a review). More recently, a variety of studies have demonstrated that changes in pupil size are linked not only to changes in luminance, but also to aspects of the sympathetic and parasympathetic nervous systems. This includes attention, mobilization, and allocation (Seropian et al., 2022), general arousal levels (Ayasse and Wingfield, 2020), task-evoked changes in arousal (Hopstaken et al., 2015), fatigue (Alhanbali et al., 2021), effortful processing (McGarrigle et al., 2017; Zhao et al., 2019), and surprisal (Zekveld et al., 2018). In linguistic research, pupillometry has gained more prominence only in the past decade, now increasingly used in research on both native and non-native language processing. A great number of studies measured pupil dilation in combination with linguistic tasks testing word and sentence language processing in either auditory mode (e.g., picture-matching tasks, VWP), sentence reading and speech production (Schmidtke, 2018). Scherger et al. (2021) used pupillometry in combination with a production and a comprehension task to investigate potential effects of early and late child bilingualism on double-object constructions in German.

With regard to sentence comprehension, pupil responses are seen to indicate processing overload modulated by syntactic complexity. Engelhardt et al. (2010) tested whether prosody alone and prosody together with visual context has an effect on the online processing of garden-path sentences: they administered two spoken language comprehension tasks to English monolingual speakers, one in which the prosody of the auditory stimuli was manipulated to mismatch the syntactic structure of the garden-path sentence and one in which the task also included pictures either matching or mismatching the intended meaning. Their findings indicate that while a prosodic mismatch tends to elicit greater pupil dilatation, hence higher processing overload during sentence comprehension, the effect of prosody is modulated when combined with visual context. Piquado et al. (2010) compared pupillary responses of younger and older English monolingual adults during a sentence listening and recall task. The study tested relative clauses manipulated by complexity (i.e., subject and object RC type) and length (with and without modifiers) to test whether processing load was modulated by syntactic complexity. While the younger group had greater pupil dilation when recalling both the more complex (object RC) and longer structures (object RC with modifiers), pupil dilation in the older group was affected only by sentence length. The authors argue that the lack of an effect of syntactic complexity in pupillary responses in the older group supports the hypothesis of “an age-specific dissociation of memory load vs. syntactic complexity effects” (Piquado et al., 2010, p. 12; see also Just and Carpenter, 1993 for a similar study).

In bilinguals, pupil responses have been shown to be modulated by the language experience of the L2 (e.g., Yao et al., 2023). Schmidtke (2014) compared the performance of monolingual and bilingual speakers of English during a word recognition task to test the effect of language experience (among other factors) on lexical retrieval efforts. Schmidtke (2014) found delayed pupil responses in bilinguals at lower level of proficiency, which was interpreted as evidence that lexical retrieval comes at a cost for bilinguals with less experience in the target language. This study is relevant to ours as it at least implicitly addresses use via the measure of experience.

The use of pupillometry in bilingualism research has also been applied to the study of code-switching in Spanish/English bilinguals. A pioneer pupillometry study comparing the online processing of single-word insertion and multi-word alternation in nominal phrases revealed a larger pupil response for the language mixing conditions compared to a unilingual baseline condition and a difference between single-word insertions and alternations in the female condition only, suggesting that the observed difference in pupil dilation is modulated by the gender of the noun (Johns and Dussias, 2022). Pupillometry as a methodology could also have a potentially positive impact in bilingual language assessment in the early diagnosis of developmental language disorders. This methodology has been used for the first time to compare sentence processing in (presumably) monolingual children already diagnosed with Specific Language Impairment (Lum et al., 2017) and proposed as an optimal tool to detect bilingual children at risk early in their linguistic development under the assumption that children with a language disorder may not show an increase in pupil dilation across grammatical and ungrammatical conditions compared to typically developing children (Scherger, 2022). Given its recent flourishing in language studies and its many applicabilities, pupillometry poses as a promising research tool to study cognitive processes in typical and atypical bilingual populations. In our study we use pupil dilation as an indicator of the increased processing load associated with ungrammaticality.

## 3. The present study: comparing group-level (L2AoA) and individual-level (usage) analyses

The conflicting group results of the AJT and pupillometry tasks in Martohardjono et al. (2021) coupled with the insights gained on the role of L2 use in L1 processing from the ERP study (Phillips et al., 2021) led us to the present study where we performed additional analyses on a subset of the data collected in the pupillometry task.^6^ In particular, we were interested in comparing group to individual level analyses, whereby the group analysis separated HS and LB by onset of bilingualism, early for HS, late for LB, while in the individual analyses use is measured as a continuous variable across all participants. Secondly, we were interested in investigating how two calculations of use, historical use over time and current use, affect processing of ungrammaticality in the L1. Based on the results of the studies summarized in section 2.2., showing that use variables significantly impact neurological, psycholinguistic, and behavioral outcomes in bilinguals, we hypothesized that relative language use would be predictive of recognition of ungrammaticality in the L1 Spanish: the greater the use of the L1, the greater the recognition of ungrammaticality as measured in relative pupil dilation. Specifically, we expect that due to increased processing load, ungrammatical items will elicit larger pupil dilation than grammatical items across the three conditions tested, wh-islands, temporal adverbial islands, and relative clause islands. However, given that wh-islands are considered weak violations, compared to the other two islands which are considered strong violations, we expect this relative weakness to be reflected in pupil size differential as well. Furthermore, we expect the ungrammatical-grammatical differential to manifest across all participants, modulated by usage. This would show that language use plays a significant role in the processing of the L1 regardless of onset of bilingualism.

### 3.1. Materials and methods

#### 3.1.1. Participants

Of the 60 participants that took part in the larger study (see section 2.3.2), data from 51 were included in this reanalysis. All were Spanish-English bilinguals between ages 18–45 (*M_Age_* = 28.02, *SD_Age_* = 7.41). To assess their eligibility, all participants completed a language history questionnaire and provided self-ratings for their comprehension fluency in Spanish on a five-point scale (*M* = 4.88, *SD* = 0.32). Because our focus was on comparing age of onset of bilingualism to use factors and because LB tend to be more L1-proficient than HS, only participants who rated their fluency as four or higher were included in the study. That is, we did not want variation in L1 proficiency to act as a confound in the design of our study. Based on age of arrival, participants who were either born in the United States or arrived in the country during early childhood were categorized as Spanish heritage speakers (HS: *N* = 30; mean age: 26; Mean AoA Spanish = 0; Mean AoA English = 4.4 (school-age) whereas those whose L2 acquisition occurred after age 15 were considered late bilinguals (LB: *N* = 21; mean age 32; Mean AoA Spanish = 0; Mean AoA English = 15 (instructed learning abroad); Mean AoArrival = 26.

#### 3.1.2. Language background questionnaire

All participants were administered a Language Background Questionnaire (LBQ) in two separate sections (see Supplementary material for the complete LBQ). The first section, based on Li et al. (2006), was administered before the experimental session and included questions about historical language background. Specifically, participants stated their native language and all languages spoken, as well as the Age of Acquisition (AoA), Context and Mode of Acquisition (i.e., where and how) and also self-rated their level of proficiency on a scale from 1 (i.e., *I have limited knowledge of the language*) to 5 (i.e., *I am a native speaker/user of the language*). Participants were asked about their first-learned language, any additional languages they were exposed to in their household while growing up, the degree of the exposure (i.e., languages most spoken), and languages used among members of the household. This first section of the questionnaire also covered questions about participants’ educational background, country of residence, and primary language(s) used in their communities and schools attended.

The second section of the LBQ was administered at the end of the experimental session and collected participants’ demographic data (i.e., sex, profession, social class) as well as data about participants’ current language use and attitudes. The items for this part were created in our lab and focused on relative language ability and use. Participants listed all the languages in which they read and write, the learning age and self-rated their reading/writing ability for each language on a scale from 1 (i.e., *I have a limited reading/writing ability in the language*) to 5 (i.e., *I am a native reader/writer of the language*). Participants were asked about their current language use preferences (i.e., *English, Spanish, Both, N/A*) with members of their family (i.e., father, mother, siblings, children, significant other), work (i.e., boss, co-workers), friends, classmates; and they quantified their use of Spanish (i.e., *mostly, little, none, N/A*) in seven different contexts (home, school work, social activities, reading, listening to the radio/music, watching TV). Participants then quantified their everyday use of both Spanish and English in percentages and specified the contexts in which the interactions typically occur. The final part of the LBQ asked about participants’ traveling practices in Spanish-speaking countries and their preferred language (English or Spanish). The LBQ was administered in English.

### 3.2. Stimuli

The stimuli analyzed for this study consisted of 3 of the 4 structures tested in the original pupillometry study (Martohardjono et al., 2021): Wh-islands, Temporal Adverbial islands, and Relative Clause islands.^7^ All stimuli sentences were recorded in Spanish by a female native speaker and created in couplets, each presenting a declarative statement as context [see example 4–6 (a)], followed by a wh-interrogative [see examples 4–6 (b) and (c)]. Different items were created for each island type in both grammatical [examples in (b)] and ungrammatical [examples in (c)] versions by questioning a noun phrase (NP) inside a syntactic island. The grammatical conditions differed from their ungrammatical counterparts in changing the questioned NP. The wh-island and temporal adverbial island each had 30 items while the relative clause island condition had 45, totaling 105 target sentences. Each ungrammatical experimental sentence was timestamped for the epoch of interest, i.e., where the ungrammaticality surfaces, whereas in the grammatical sentences, the timestamp was located at the point where the structure of interest begins. The sample stimuli indicate these boundaries with “||.” All participants were presented both the grammatical and ungrammatical versions of each item.

4)  Wh*-*island

        a.  Ignacio confirmó                                      por qué                    la enfermera

            Ignacio confirm.PRET.3SG                       why                         the nurse

            había                    llevado                           la medicina.

            have.IMP.3SG     bring.PART                    the medicine

            ‘Ignacio confirmed why the nurse had brought the medicine.’

        b.  ¿Qué enfermera confirmó                        Ignacio || que

            what nurse            confirm.PRET.3SG      Ignacio                    COMP

            había                     llevado                         la medicina?

            have.IMP.3SG      bring.PART                  the medicine

            ‘What nurse did Ignacio confirm had brought the medicine?’

        c.  *¿Qué enfermera confirmó                      Ignacio || por qué

            what nurse            confirm.PRET.3SG      Ignacio                    why

            había                     llevado                         la medicina?

            have.IMP.3SG      bring.PART                  the medicine

            ‘What nurse did Ignacio confirm why had brought the medicine?’

5)  Temporal adverbial island

        a.  El niño comió              el dulce mientras que                          su tía

            the child eat.PRET.3SG the candy  while  COMP his aunt

            buscaba                                                      la comida.

            search.IMP.3SG                                         the food

            ‘The child ate the candy while his aunt looked for food.’

        b.  ¿Qué niño comió            el dulce || mientras que                    su

            what child eat.PRET.3SG the candy          while      COMP his

            tía buscaba                         la comida?

            aunt search.IMP.3SG         the food

            ‘What child ate the candy while his aunt looked for food?’

        c.  *¿Qué tía || el niño comió                       el dulce mientras

            what aunt the child            eat.PRET.3SG the candy while

            que buscaba                       la comida?

            COMP search.IMP.3SG    the food

            ‘What aunt did the child eat the candy while looked for food?’

6)  Relative clause island

        a.   Paola hizo                    el gesto que        causó

            Paola make.PRET.3SG the joke COMP    cause.PRET.3SG

            la controversia

            the controversy

            ‘Paola made the joke COMP caused the controversy.’

        b.  ¿Qué gesto hizo             Paola || que             causó

            what joke make.PRET.3SG Paola COMP      cause.PRET.3SG

            la controversia?

            the controversy.

            ‘What joke did Paola make that caused the controversy?’

        c.  *¿Qué controversia hizo                             Paola || el gesto

            what controversy make.PRET.3SG              Paola the joke

            que      causó?

            COMP cause.PRET.3SG

            ‘What controversy did Paola make the joke that caused?’

### 3.3. Procedure

Stimuli sentences were presented in the aural modality given its suitability for heritage speakers. In each trial, the context sentence was followed by the target sentence, and trials were pseudorandomized over five blocks. Throughout the auditory blocks, participants fixated their gaze on a white “+” marker centered on a black screen. To ensure task engagement, yes/no comprehension probes followed 40% of the trials.^8^ Participants read the task instructions in the language of preference (Spanish or English) and were given a practice block to familiarize themselves with the task.

Tobii TX300 infrared cameras were used to record the pupil diameter and gaze location for each eye separately. Data were gathered at 60 Hz for the whole trial (one sample every 16.67 milliseconds) during both the context and target sentences, as well as for the preceding and following 1,000 ms before and after each trial.

### 3.4. Analysis

#### 3.4.1. Pre-processing

For each trial, any samples that were marked as invalid during recording (a Tobii validity code of 1, 2, 3, or 4) were excluded; this includes the pupil diameter and x- and y-gaze positions for both the left and right eyes. Missing samples were not interpolated as interpolation can increase autocorrelation in the residuals leading to anti conservative models (see van Rij et al., 2019, p. 5). Next, the pupil diameter and x- and y-gaze positions were averaged for the left and right eyes. Data were time-locked to the point of ungrammaticality (and the corresponding position in each grammatical counterpart) with the epoch of analysis extending 2,000 ms (120 samples) from this point. This 2,000-ms window was chosen for two reasons: First, since the onset of the epoch was unique for each sentence, the duration of the epoch was also variable. This time window ensured that >90% of all trials had data up to this point. Second, 2,000 ms was determined to be sufficient to capture the task-evoked pupil response, given previous research that suggests that the pupillary response emerges roughly 500 to 1,500 ms post-stimulus onset (Hoeks and Levelt, 1993; Winn et al., 2015, 2018; Winn, 2016). The average pupil size was calculated during the 200-ms (12-sample) period before the onset of this epoch, and baseline subtraction was performed to account for non-stimulus-related changes in pupil size during the course of the experiment. Trials where more than 35% of all samples were marked for exclusion were removed,^9^ resulting in 37% of all trials being removed. Participants with an insufficient number of trials within each structural condition were likewise excluded from the analysis for that particular condition only (wh-island: 14 participants; temporal adverbial: 13 participants; relative clause: 9 participants).

#### 3.4.2. Generalized additive mixed models

Data were analyzed using generalized additive mixed-effects models (GAMMs) using the bam function in the *mgcv* package (v. 1.8-33; Wood, 2011; Wood et al., 2016) with further model criticism and visualization performed using the *itsadug* package (v. 2.3; Van Rij et al., 2020). GAMMs are ideal for analyzing time-series data, like the task-evoked pupil response (TEPR), as it is able to capture non-linear dependencies in the data as well as account for autocorrelation using an embedded autoregressive (AR-1)—an added benefit over using other modeling techniques such as growth curve analysis. Data for each of the island types was analyzed separately, but all followed the same procedure (see Supplementary material for the full analysis scripts). First, a maximally specified reference model was fit without the inclusion of an embedded AR1 model in order to determine the appropriate value for the autocorrelation coefficient *rho*, which was extracted using the start_value_rho function in the *itsadug* package. Next, the model was re-run with an embedded AR1 model with this specified *rho* value. The acf_resid function in the *itsadug* package was used to ensure that autocorrelation in this final model was within acceptable levels; if not, the *rho* value was manually adjusted until the autocorrelation at lag 1 was sufficiently low (<0.2). The gam.check function in the *itsadug* package was used to determine the appropriate number of knots, *k*, for each smooth term in the model. All models were specified to use a scaled-*t* distribution to account for the non-normal distribution of the data. Time was entered into the model as the sample number, which was re-numbered such that sample 1 was the first sample that corresponded to the start of the epoch of analysis. Given that the epoch extended for 2,000 ms and each sample was approximately 16.67 ms, the total number of samples for the epoch of analysis was 120. In all models, a smooth term for gaze position was included to account for its effects on pupil size (Gagl et al., 2011). This smooth term modeled the x- and y-gaze position as a continuous, non-linear interaction, allowing for the effects of gaze position on pupil size to be modeled directly as a covariate. Lastly, random smooths by participant and by item were included as well (van Rij et al., 2019).

For each island type, two different models were run. The first was a binary coded model (see Wieling, 2018) that estimated the differences between the two groups (LB, HS) and the two conditions (grammatical, ungrammatical) as well as the interaction between them. Given that binary-coded variables represent specific contrasts within the model, this model was subsequently releveled—in the same way that a linear model might be releveled—to examine all contrasts of interest. The comparisons of interest were:

1. LB, ungrammatical minus LB, grammatical
2. HS, ungrammatical minus HS, grammatical
3. HS, grammatical minus LB, grammatical
4. HS, ungrammatical minus LB, ungrammatical
5. The difference in the grammaticality effect between HS and LB.

**Figure 1:**
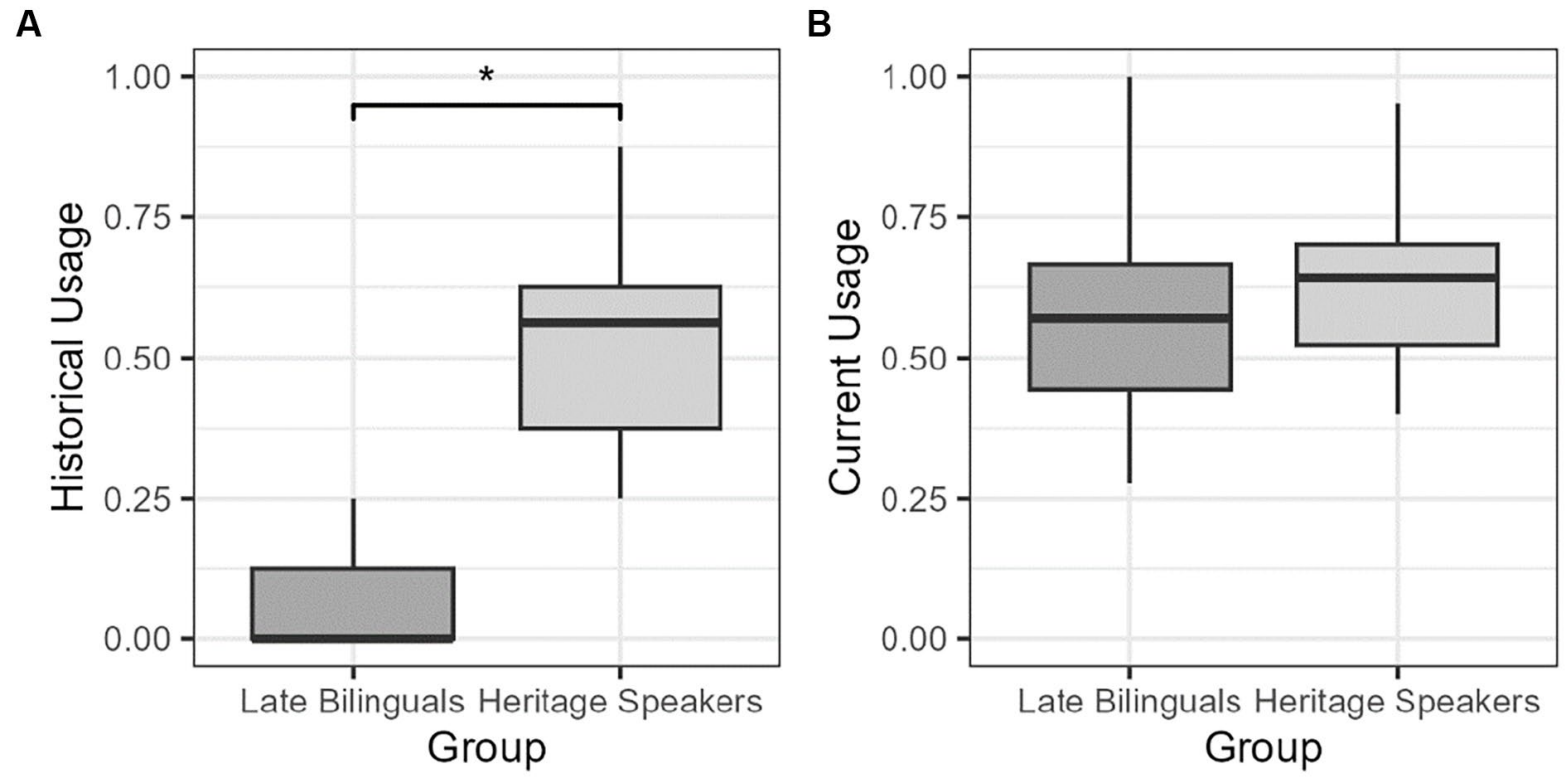
**(A)** Historical and **(B)** current usage of Spanish by group.*p < 0.05.

The second model sought to examine the grammaticality effect not as a function of group but rather as a function of current and historical usage of Spanish. Both usage variables were continuous predictors derived from different questions in the LBQ. Current usage was derived from the following question and its subcomponents: “How much Spanish do you use in/at: 1) home, 2) school, 3) work, 4) social activities, 5) reading, 6) listening to the radio/music, and 7) watching TV?.” Possible answers were “Mostly,” “Both” (meaning both Spanish and English in equal amounts), “Little,” “None,” and “Not Applicable” (which was excluded). These answers were converted to numeric values (3, 2, 1, and 0, respectively), the average was taken across the seven domains in the question, and the value was rescaled between 0 and 1, where 0 indicated “exclusively Spanish” and 1 indicated “no Spanish.” Historical usage was derived from the following questions: “What languages were spoken in your house growing up?,” “Which of the languages from [the previous question] were used most often?,” “What was the primary language spoken in your local community?,” and “What was the language of instruction?” Possible answers were “Spanish,” “Both Spanish and English,” and “English.” These answers were converted to numeric values (0, 0.5, and 1) respectively and the average was taken across these four questions such that 0 indicated “exclusively Spanish” and 1 indicated “exclusively English.” Two-sample t-tests revealed that, while there was a significant difference in historical usage between the two groups (*t* = −10.1, *p* < 0.001, Figure 1A), there was no difference in current usage between the two groups (*t* = 1.11, *p* = 0.27; Figure 1B). This shows that language use over time separates the late bilinguals from heritage speakers, with LB having more Spanish use, while current use of both Spanish and English overlaps between the two groups.

To model current and historical usage as continuous predictors, they were included as two decomposed tensor product interactions, which allowed pupil size to be modeled not only as a non-linear function of time but also as a non-linear function of usage. This way, it is possible to determine how each term modulates the grammaticality (coded as a binary variable) effect in each of the three island types. Likewise, both current and historical usage were included in the same model so they could be compared against each other directly while also controlling for the other. For example, if the interaction between current usage and grammaticality is significant, but the interaction between historical usage and grammaticality in that same model is non-significant, it suggests that the former is a better predictor of the grammaticality effect even when the latter is taken into account. Lastly, a significant interaction term indicates that the effect of the usage variable on the grammaticality effect is significantly ‘wiggly’, that is, has a non-zero and non-linear effect on the pupil size as it changes over time. Given that the two models for each island type were non-nested, model comparison was not performed. All figures below are model estimates plotted using the *itsadug* package. R code for all of the analyses and visualizations below can be found in Supplementary material.

### 3.5. Results

#### 3.5.1. Wh-islands: group differences in the grammaticality effect

**Figure 2:**
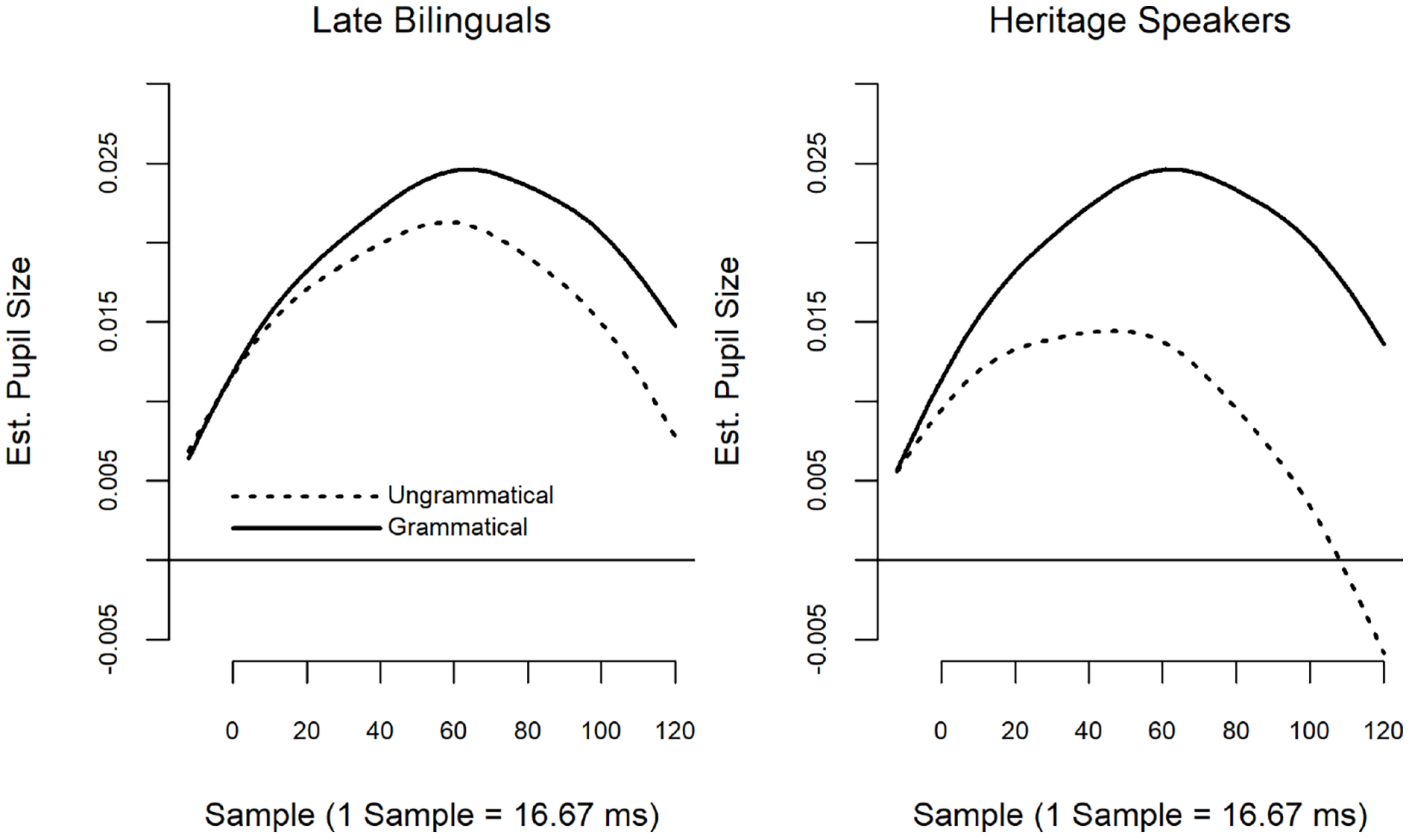
Wh-islands fitted smooths: group by grammaticality.

The summary of the model with LB, Grammatical as the reference level is provided in Table 1; summary tables of the model when revealed are provided in Supplementary material. Fitted smooths are presented in Figure 2. Model summary tables present the binary-coded difference smooths, represented by terms beginning with ‘Is’, and indicate whether a given difference smooth is significantly different from zero. Difference smooths are always compared back to the reference level, represented by “s(Sample),” which is congruent to the intercept in a linear model. For example, in Table 1, the term ‘s(Sample)’ represents the fitted smooth for late bilinguals (LB) in the grammatical condition. The second term, “IsUngram,” then estimates the difference smooth between ungrammatical and grammatical items for LB; that is, when the only change vis-à-vis the reference level is from grammatical to ungrammatical. Interaction terms (“IsUngramHS”), through the same logic, represent the difference in the grammaticality effect (ungrammatical minus grammatical) between the two groups.

**Table 1:** Wh-islands model summary (reference: LB, grammatical).

Parametric coefficients	β	SE	*t*	*p*	
(Intercept)	0.00	0.00	−0.36	0.72	
					
Smooth terms	EDF	Ref.DF	*f*	*p*	
s(Sample)	3.92	4.74	5.34	<0.001	*
s(Sample): IsUngram	2.01	2.01	1.79	0.18	
s(Sample): IsHS	2.57	2.80	0.03	0.92	
s(Sample): IsUngramHS	2.01	2.01	3.48	0.02	*
s(X Gaze, Y Gaze)	37.19	38.80	91.72	<0.001	*
s(Sample, Subject)	174.89	458.00	1.76	<0.001	*
s(Sample, Item)	37.07	299.00	0.64	<0.001	*

*p < 0.05.

The model suggested that the two groups did not differ from one another in neither the grammatical (*F* = 0.03, *p* = 0.99) nor ungrammatical (*F* = 0.66, *p* = 0.58) conditions. However, there was a significant interaction between Group and Grammaticality (*F* = 3.48, *p* = 0.03) such that HS showed a significant difference between the grammatical and ungrammatical items (*F* = 14.03, *p* < 0.001) while LB did not (*F* = 1.79, *p* = 0.19). However, the effect was in the opposite direction from that expected: grammatical items elicited *larger* pupillary responses than ungrammatical items.

#### 3.5.2. Wh-islands: effects of current and historical usage

**Figure 3:**
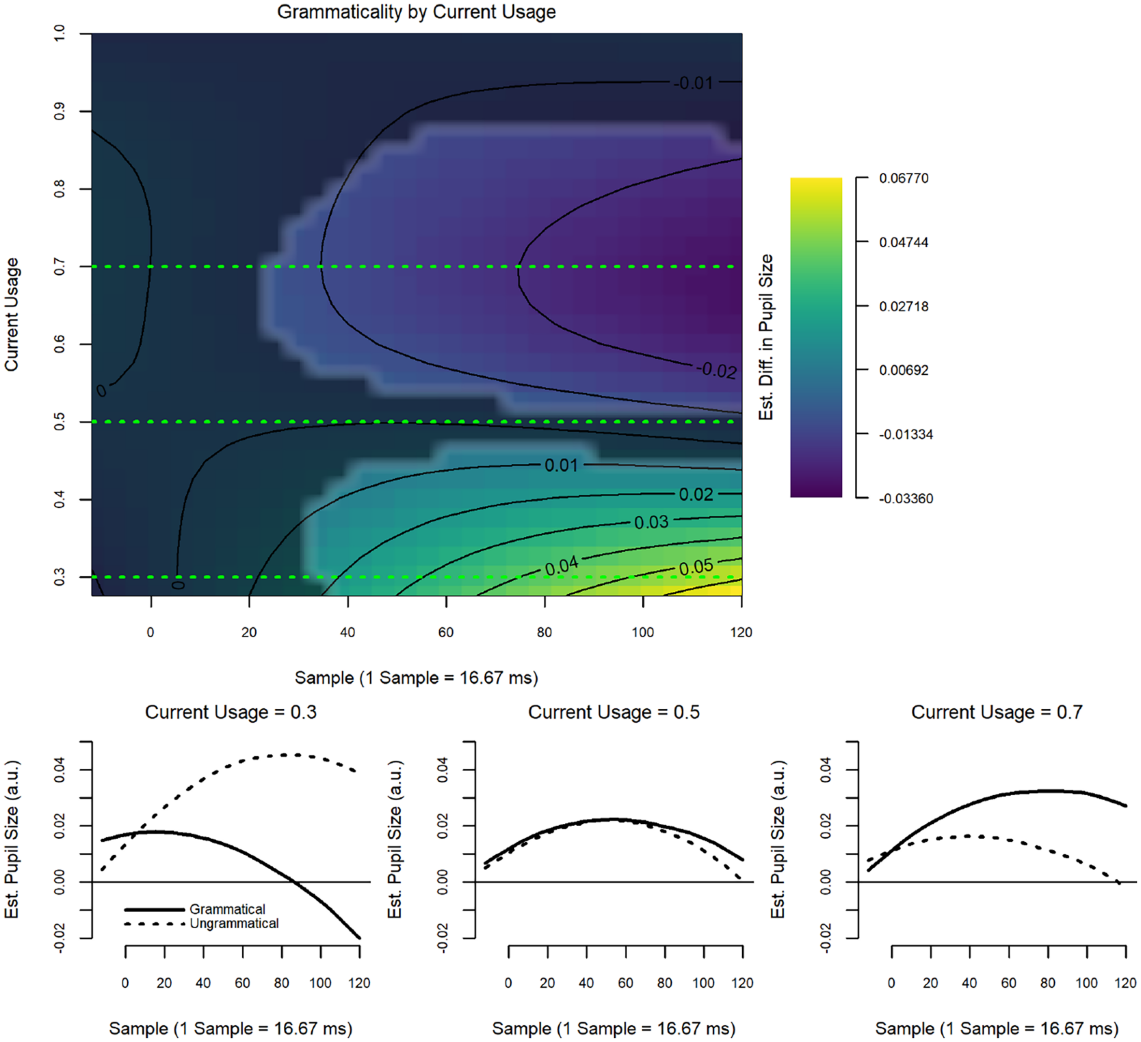
Wh-islands: current usage by grammaticality.

The model revealed a significant interaction between current usage and grammaticality (*F* = 4.35, *p* < 0.001; Figure 3), but the interaction between historical usage and grammaticality was non-significant (see Table 2 for model summary). Figure 3 provides the heatmap showing the estimated strength of the grammaticality effect as a function of current usage of Spanish; that is, the difference of ungrammatical minus grammatical, where positive values indicate larger pupil sizes in response to ungrammatical vs. grammatical items. This is also indicated by the coloration: warmer colors indicate a larger positive difference, while cooler colors indicate a smaller (or negative) difference. The x-axis shows the time into the trial, with 0 corresponding to the onset of the epoch. The y-axis displays the usage variable, with lower values indicating more usage of Spanish and higher values indicating more usage of English. The other three panels present ‘slices’ of the heatmap at different values of Current Usage (noted in the titles), showing the pupillary responses to grammatical and ungrammatical items at these values. More current usage of Spanish (lower values) was associated with a strong grammaticality effect, with ungrammatical items eliciting larger pupil sizes than grammatical items. More current usage of English (higher values), however, was associated with a *reverse* grammaticality effect, with grammatical items eliciting larger pupil sizes than ungrammatical items.

**Table 2:** Wh-islands model summary: usage by grammaticality.

Parametric coefficients	β	SE	*t*	*p*	
(Intercept)	0.0002	0.0035	0.07	0.94	
					
Smooth terms	EDF	Ref.DF	*f*	*p*	
s(Sample)	3.88	4.63	5.36	<0.001	*
s(Sample): IsUngram	1.00	1.01	0.36	0.55	
s(Historical Usage)	1.00	1.00	1.52	0.22	
s(Historical Usage): IsUngram	1.01	1.01	1.87	0.09	
s(Current Usage)	1.40	1.46	1.99	0.10	
s(Current Usage): IsUngram	3.79	4.26	7.88	<0.001	*
ti(Sample, Historical Usage)	1.01	1.02	1.18	0.28	
ti(Sample, Historical Usage): IsUngram	2.32	2.79	1.06	0.27	
ti(Sample, Current Usage)	2.04	2.19	2.93	0.05	
ti(Sample, Current Usage): IsUngram	4.35	5.80	4.20	<0.001	*
s(X Gaze, Y Gaze)	37.73	38.90	122.36	<0.001	*
s(Sample, Subject)	159.47	387.00	1.43	<0.001	*
s(Sample, Item)	65.15	299.00	0.38	<0.001	*

*p < 0.05.

#### 3.5.3. Temporal adverbial islands: group differences in the grammaticality effect

**Figure 4:**
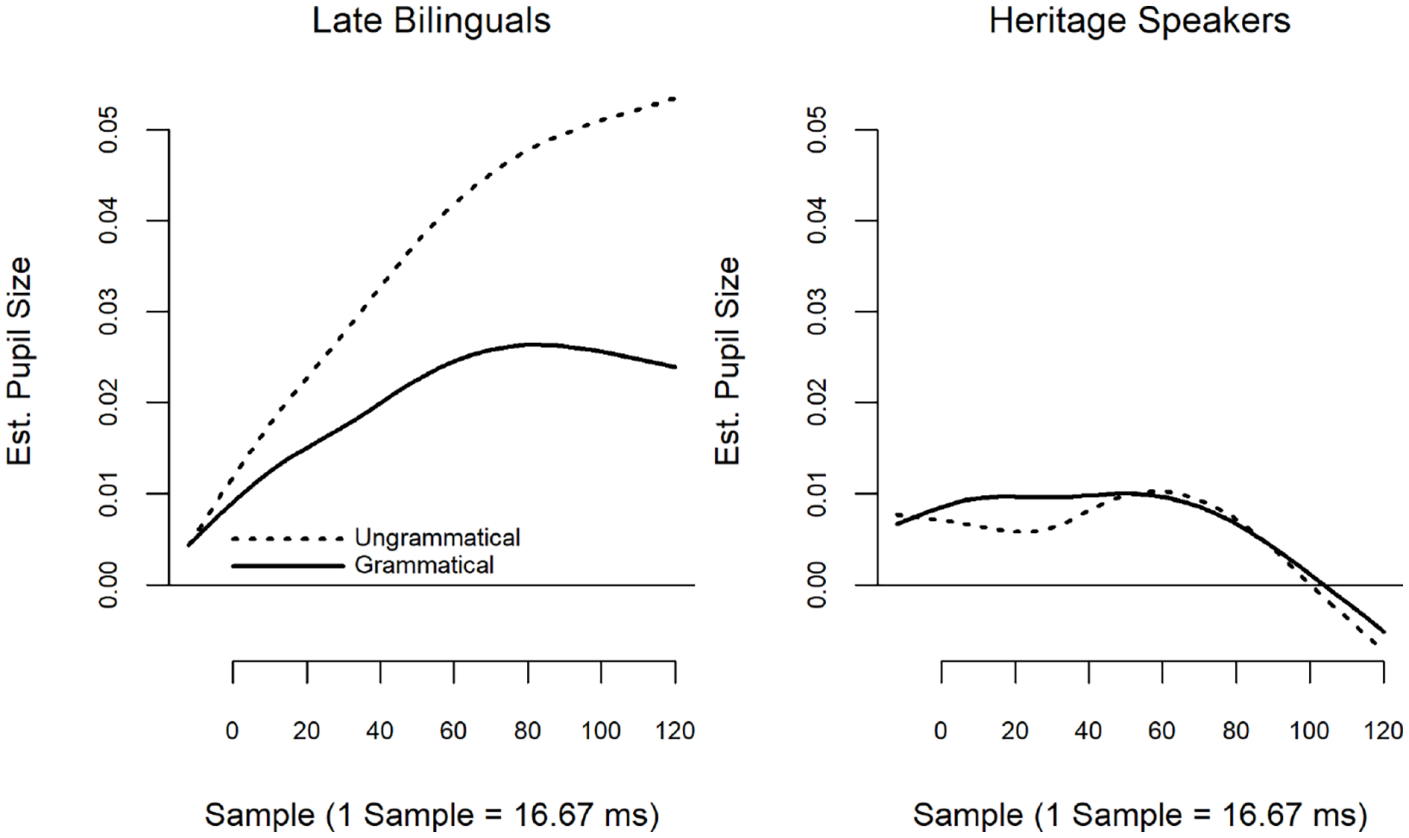
Temporal adverbial islands: group by grammaticality.

The summary of the model with LB, Grammatical as the reference level is provided in Table 3; summary tables of the model when releveled are provided in Supplementary material. Fitted smooths are presented in Figure 4. The model revealed a significant interaction between Group and Grammaticality (*F* = 8.74, *p* < 0.001). LB showed a significant effect of grammaticality, with ungrammatical items eliciting larger pupillary responses than grammatical items (*F* = 20.53, *p* < 0.001). There was no difference between grammatical and ungrammatical items for the HS.

**Table 3:** Temporal adverbial islands model summary (reference: LB, grammatical).

Parametric coefficients	β	SE	*t*	*p*	
(Intercept)	−0.19	0.00	−4.72	<0.001	*
					
Smooth terms	EDF	Ref.DF	*f*	*p*	
s(Sample)	3.24	3.78	3.51	0.01	*
s(Sample): IsUngram	2.35	2.58	20.53	<0.001	*
s(Sample): IsHS	2.01	2.01	7.05	<0.001	*
s(Sample): IsUngramHS	3.45	4.08	8.74	<0.001	*
s(X Gaze, Y Gaze)	38.57	38.99	307.93	<0.001	*
s(Sample, Subject)	208.83	469.00	1.72	<0.001	*
s(Sample, Item)	105.26	300.00	0.96	<0.001	*

*p < 0.05.

#### 3.5.4. Temporal adverbial islands: effects of current and historical usage

**Figure 5:**
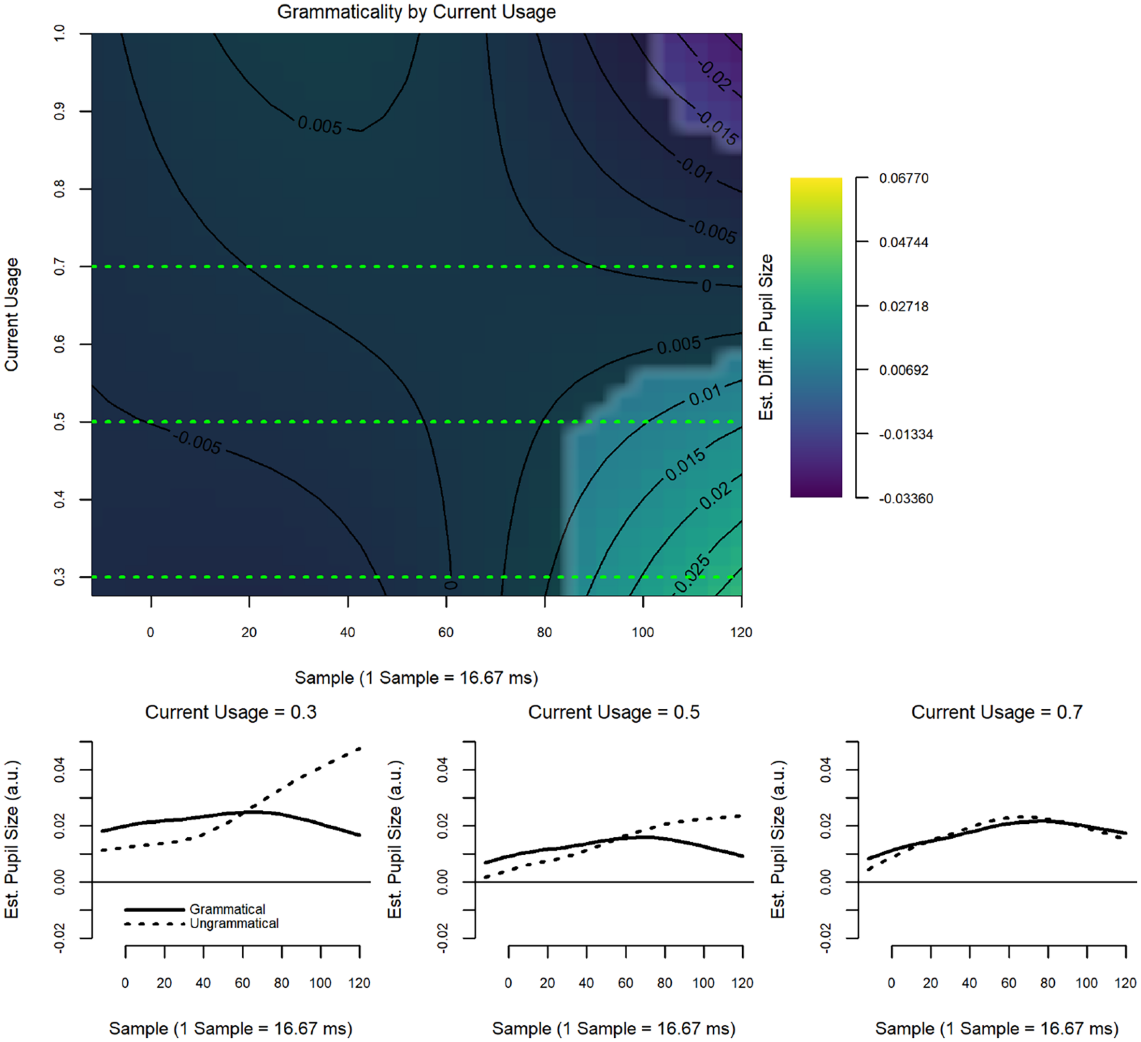
Temporal adverbial islands: current usage by grammaticality.

**Figure 6:**
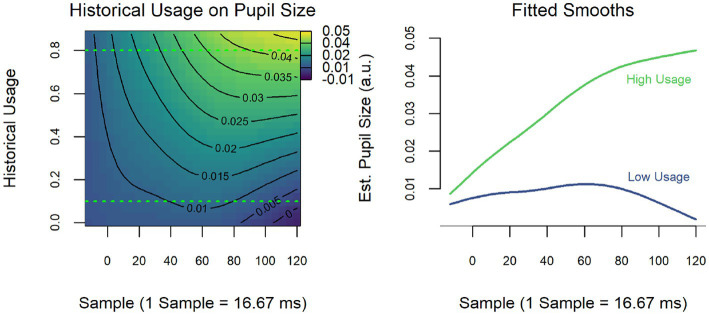
Temporal adverbial islands: historical usage of Spanish on pupil size.

The model revealed a significant interaction between current usage and grammaticality (*F* = 3.48, *p* = 0.02; Figure 5), but the interaction between historical usage and grammaticality was non-significant (see Table 4 for model summary). Nonetheless, historical usage did have an overall effect on pupil size that did not differ based on grammaticality (*F* = 4.93, *p* = 0.03; Figure 6): decreasing historical use of Spanish (i.e., higher values) are associated with overall larger pupillary responses. As for the interaction between current usage and grammaticality, individuals who reported more current use of Spanish showed a strong, late grammaticality effect, while those who reported more current use of English showed a small reversal of this effect late in the epoch.

**Table 4:** Temporal adverbial islands model summary: usage by grammaticality.

Parametric coefficients	β	SE	*t*	*p*	
(Intercept)	−0.02	0.00	−6.05	<0.001	*
					
Smooth terms	EDF	Ref.DF	*f*	*p*	
s(Sample)	3.02	3.55	1.63	0.16	
s(Sample): IsUngram	2.00	2.00	1.59	0.20	
s(Historical Usage)	1.00	1.00	7.81	0.01	*
s(Historical Usage): IsUngram	2.91	3.31	2.52	0.05	
s(Current Usage)	2.72	2.79	1.35	0.16	
s(Current Usage): IsUngram	1.00	1.01	4.74	0.02	*
ti(Sample, Historical Usage)	1.01	1.02	4.93	0.03	*
ti(Sample, Historical Usage): IsUngram	1.03	1.05	0.50	0.51	
ti(Sample, Current Usage)	2.23	2.37	1.69	0.34	
ti(Sample, Current Usage): IsUngram	2.39	2.92	4.04	0.01	*
s(X Gaze, Y Gaze)	38.40	38.98	267.25	<0.001	*
s(Sample, Subject)	164.96	398.00	1.37	<0.001	*
s(Sample, Item)	91.57	299.00	0.71	<0.001	*

*p < 0.05.

#### 3.5.5. Relative clause islands: group differences in the grammaticality effect

**Figure 7:**
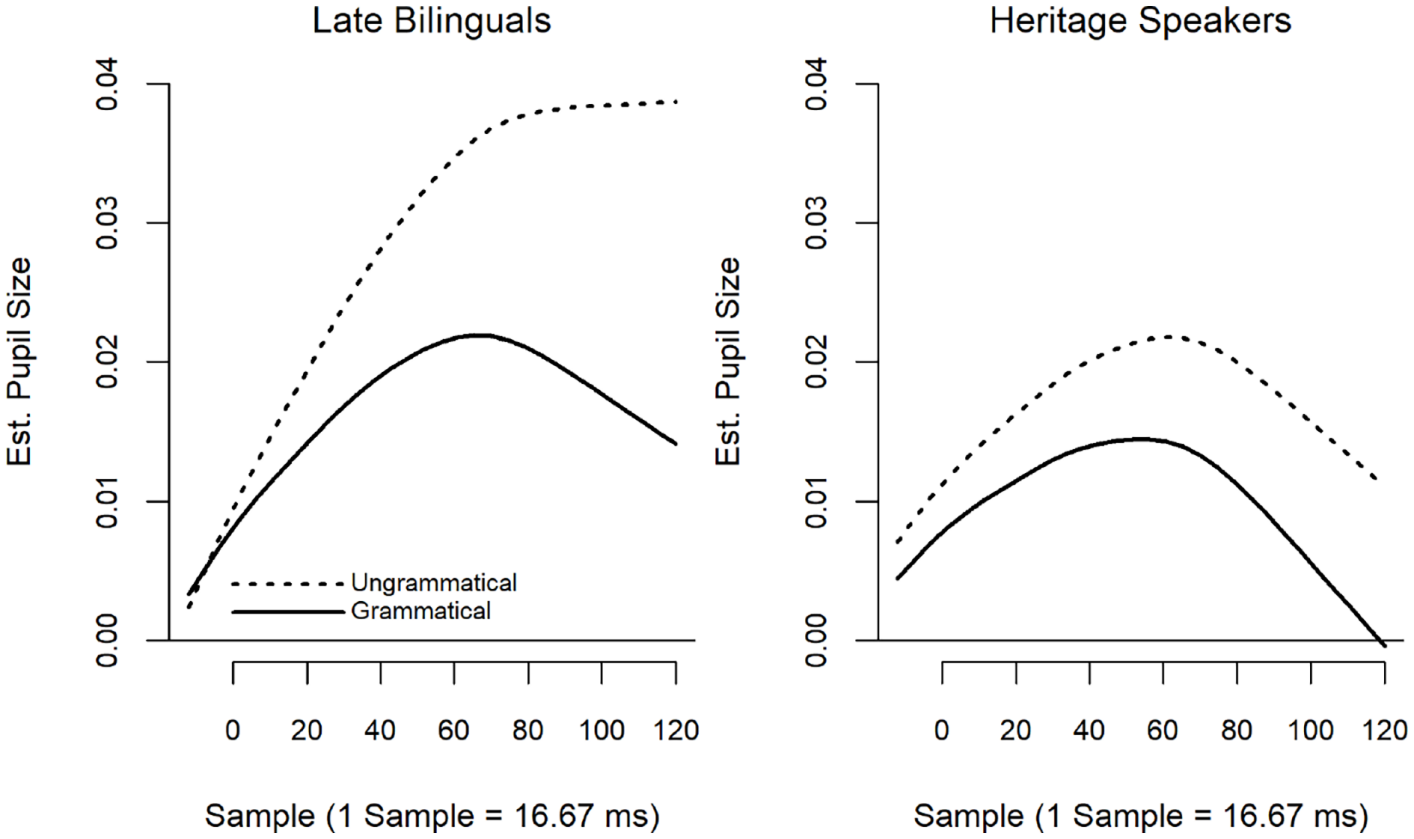
Relative clause islands fitted smooths: group by grammaticality.

The summary of the model with LB, Grammatical as the reference level is provided in Table 5; summary tables of the model when releveled are provided in Supplementary material. Fitted smooths are presented in Figure 7. The model revealed a significant interaction between Group and Grammaticality (*F* = 3.95, *p* = 0.02). While both the LB (*F* = 26.96, *p* < 0.001) and HS (*F* = 9.16, *p* < 0.001) showed a significant effect of grammaticality, with ungrammatical items eliciting larger pupillary responses than grammatical items, this effect was larger for the LB than the HS.

**Table 5:** Relative clause islands model summary (Ref: LB, grammatical).

Parametric coefficients	β	SE	*t*	*p*	
(Intercept)	−0.02	0.00	−5.98	0.00	*
					
Smooth terms	EDF	Ref.DF	*f*	*p*	
s(Sample)	3.87	4.50	5.71	<0.001	*
s(Sample): IsUngram	2.01	2.01	26.96	<0.001	*
s(Sample): IsHS	2.01	2.02	2.20	0.11	
s(Sample): IsUngramHS	2.01	2.02	3.95	0.02	*
s(X Gaze, Y Gaze)	38.49	38.98	499.95	<0.001	*
s(Sample, Subject)	238.47	508.00	2.23	<0.001	*
s(Sample, Item)	169.52	449.00	1.08	<0.001	*

*p < 0.05.

#### 3.5.6. Relative clause islands: effects of current and historical usage

**Figure 8:**
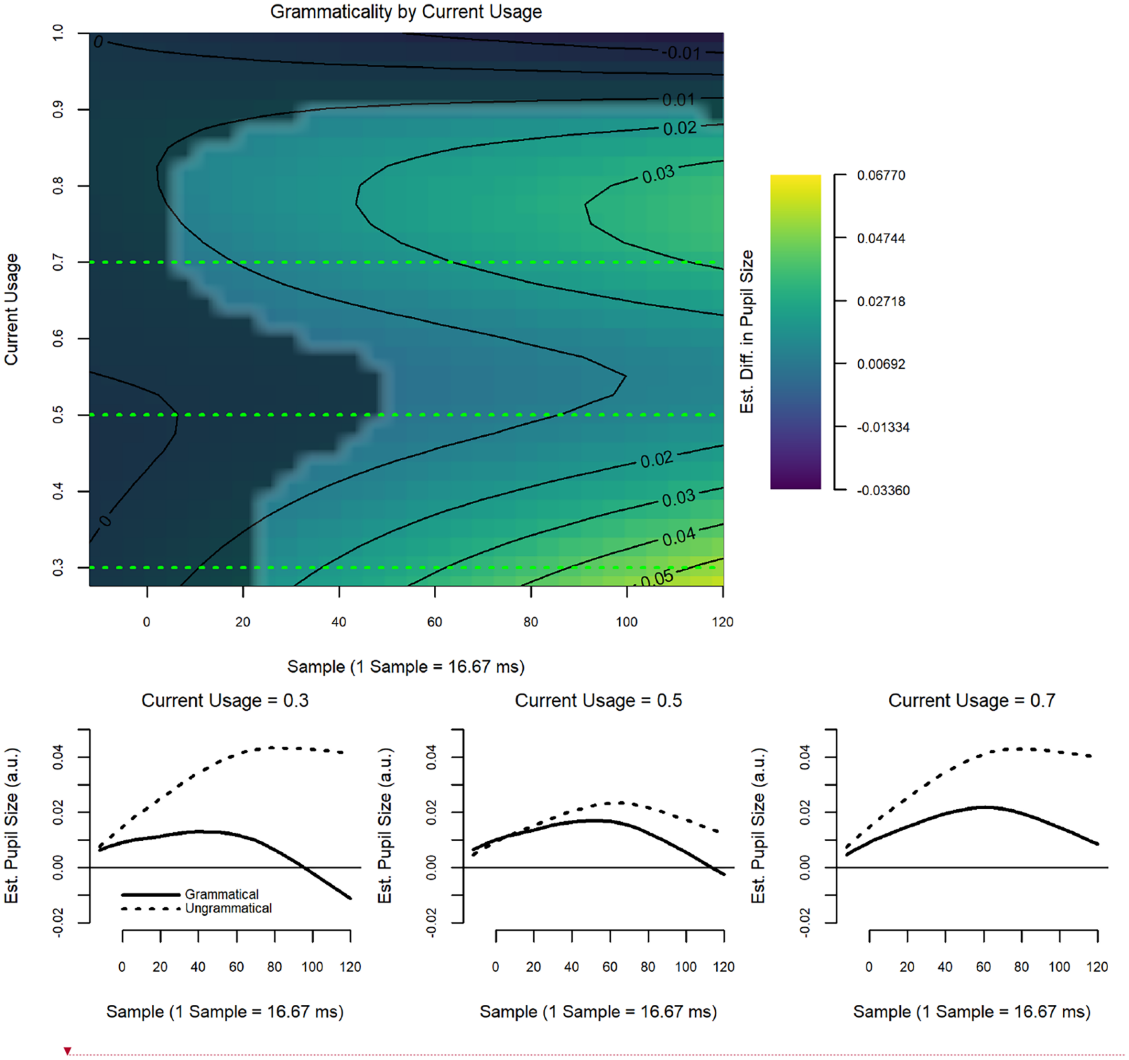
Relative clause islands: current usage by grammaticality.

The model revealed a significant interaction between current usage and grammaticality (*F* = 2.52, *p* = 0.03; Figure 8), but the interaction between historical usage and grammaticality was non-significant (see Table 6 for model summary). In this case, individuals who reported higher current usage of Spanish than English *and* individuals who reported higher usage of English than Spanish both showed late grammaticality effects in the pupillary response. Individuals who reported roughly equal amounts of Spanish and English showed little-to-no differences between the grammatical and ungrammatical conditions.

**Table 6:** Relative clause islands model summary: usage by grammaticality.

Parametric coefficients	β	SE	*t*	*p*	
(Intercept)	−0.02	0.00	−7.20	<0.001	*
					
Smooth terms	EDF	Ref.DF	*t*	*p*	
s(Sample)	4.11	4.82	6.06	<0.001	*
s(Sample): IsUngram	2.00	2.02	6.77	<0.001	*
s(Historical Usage)	1.01	1.01	0.80	0.37	
s(Historical Usage): IsUngram	1.44	1.50	0.34	0.67	
s(Current Usage)	1.00	1.00	1.51	0.22	
s(Current Usage): IsUngram	4.21	4.54	4.73	<0.001	*
ti(Sample, Historical Usage)	1.01	1.02	2.13	0.14	
ti(Sample, Historical Usage): IsUngram	1.02	1.03	2.76	0.10	
ti(Sample, Current Usage)	1.45	1.57	1.91	0.09	
ti(Sample, Current Usage): IsUngram	3.44	3.98	2.52	0.03	*
s(X Gaze, Y Gaze)	38.43	38.98	524.52	<0.001	*
s(Sample, Subject)	179.76	407.00	1.96	<0.001	*
s(Sample, Item)	145.06	449.00	0.81	<0.001	*

*p < 0.05.

## 4. Discussion

A comparison of the group-based and the usage-based models yielded divergent results across the 3 island types. We discuss these separately for each type:

For wh-islands, considered a “weak” island violation in the syntactic literature (e.g., Torrego, 1984) the model comparing HS to LB as a group did not detect differences in the way either grammatical or ungrammatical items were treated. However, although the interaction between Group and Grammaticality was significant, neither HS nor LB showed patterns that follow predictions of (un)grammaticality. For the HS, the grammatical items elicited significantly larger pupil dilation than ungrammatical ones; for the LB the difference between grammatical and ungrammatical items, while similar to the pattern seen for the HS, did not result in significance (see Figure 2). This would indicate that neither group perceived the ungrammaticality of wh-island violations. In contrast, the usage-based model showed a significant effect for current, though not for historical use. Moreover, in the usage-based model, the effect was seen in the expected direction, i.e., ungrammatical sentences eliciting larger pupil dilation than grammatical sentences. As illustrated in the heat map and slice diagrams, pupil dilation to ungrammatical Spanish sentences was modulated by whether Spanish or English was used more. As expected, more current Spanish use elicited greater pupil dilation for ungrammatical items, indicating greater processing load and greater sensitivity to Spanish ungrammaticality. Conversely, more current English use elicited smaller pupil dilation to ungrammatical items, indicating less sensitivity to Spanish ungrammaticality. This reversal is reminiscent of what was found in the ERP study (Phillips et al., 2021) where overall increased English use was inversely related to N400 amplitude to Spanish ungrammaticality. The fact that divergent results were obtained in the two models points to the importance of looking at data from different angles, in this case, both with a group as well as an individual-level analysis.

For the Temporal Adverbial islands, considered a strong violation in the syntactic literature (here indicated by **), the group model showed the expected effect for LB but not HS. This would indicate that HS are not sensitive to ungrammatical sentences like 7.

7)  **Que. tía_1_ el niño comió el dulce mientras que ____i_ buscaba la comida?

** *What aunt did the boy eat the candy while ___ looked for food?*

On the other hand, when grammaticality is examined via usage variables, we see again that current, but not historical usage is predictive of sensitivity to ungrammatical TA islands. Similar to what we saw in the wh-islands, the heat map and “slice” diagrams showed that greater current use of Spanish elicits a pupil response to these ungrammatical sentences. This effect is again reversed with increased English use where grammatical items elicit greater dilation than ungrammatical ones. Here again, the usage-based results for the TA islands align with the ERP results in Phillips et al. (2021) and stand in contrast to the results from the group-based model, which suggested no sensitivity to strong L1 ungrammaticality for the HS group. Different from the wh-island results, the usage-based model for TA islands did show an effect for historical usage, which was, however, independent of grammaticality but modulated by decreased use of Spanish over time. This suggests that less Spanish use over the lifetime incurred greater processing load for Spanish sentences containing temporal adverbial clauses overall.

Finally, for RC islands, which in the syntactic literature are considered the strongest island violation, the group model showed a grammaticality effect for both LB and HS, although this effect was significantly larger for LB than for HS. From this, one might conclude that HS are less sensitive to these strong violations than are LB. As in the case of TA-island violations, this would suggest a qualitative difference in the way L1 ungrammaticality is processed by HS, compared to LB.

Results from the usage-based model, on the other hand, revealed again that use is a significant variable, modulating detection of ungrammaticality. Here, as for the other two types, current, though not historical usage was predictive of increased pupil dilation for RC island violations. However, in this case, this was true for both more Spanish as well as more English use. While the result for more Spanish is expected, the result for more English is puzzling. A possible interpretation could be that this is a consequence of the strength of this type of violation. Questioning a noun located inside a relative clause (el crítico in 8a below) arguably results not only in a strong violation but in a virtually unparsable and therefore uninterpretable structure (seen in b).

8)  Declarative

      a.   El cine mostró el documental que **el crítico** odiaba.

        ‘The cinema showed the documentary that **the critic** hated.’

  Question:

      b. ** ¿Qué crítico mostró el cine el documental que ___odiaba.

        ‘Which critic did the cinema show the documentary that___hated?’

Considering that this is true in both Spanish and English, it may be the case that the uninterpretability of such sentences requires increased processing effort regardless of which language is used more, which is what could be reflected in the heat maps and diagrams. What is important for our purposes, is that this was true for individuals in both the HS group and the LB group.

Finally, how can the different results between current and historical use be interpreted? Historical use was measured by questions that asked about language use over the lifetime. As shown in Figure 1A, historical use separates LB participants from HS participants, as would be expected, since onset of bilingualism occurs later for LB than for HS. That is, LB use Spanish more over their lifetime than do HS over theirs. Current use, on the other hand, was measured by questions related to language use at and around time of testing. Figure 1B shows that there was overlap between HS and LB in the use of Spanish and English. The result we obtained showing that historical use does not play a role in the detection of ungrammaticality, while current use does, suggests that recent use of the HL affects sensitivity to ungrammaticality, while cumulative use does not.

## 5. Conclusion

We began this paper with the observation that research on Heritage Speakers has typically labeled these bilinguals as being distinct from other bilinguals, a characterization that is primarily based on age of acquisition (of the L2), L2 dominance, and group analyses. This separation into type and group, we have argued, largely ignores the heterogeneity that must necessarily hold across all bilingual speakers. This heterogeneity might even be greater for HS than for LB, given the greater linguistic and societal experiences HS encounter. It is reasonable to assume that the great variability and large number of factors, linguistic and extra-linguistic, influencing the bilingual experience of HS should defy attempts of strict categorization of this population, at least from a cognitive perspective.^10^ At the same time, the heterogeneity of experiential factors determining the bilinguality of the HS individual would lend itself better to a perspective that views that individual as being on a continuum. While it is impossible to address all or even the majority of these factors in an empirical study, we noted that usage variables have largely been ignored in the HS literature, even as there is increasing evidence of its significance in the general bilingual literature. We have therefore chosen to use the continuum of usage in our analysis. Furthermore, the literature has often ignored the inclusion of fluent HS populations and by doing so has risked confounding the effect of age of L2 exposure, L2 dominance and heritage language proficiency. Fluent HS are abundant in areas where there is a vibrant community speaking the HL. We sought to address these issues by comparing exclusively fluent HS and LB and focusing on variables of relative L1 (Spanish) and L2 (English) use, both historical and current. We chose an implicit method that is gaining increased use in experimental studies of language, pupillometry, on the detection of ungrammaticality in the L1 because of its fine temporal resolution and its ability to provide moment by moment data while at the same time being less invasive than neurophysiological methods such as EEG.

Our findings indicate that current use of the L1 Spanish plays a significant role in the detection of ungrammaticality in that language. Specifically, with greater current use of the L1, both weak and strong violations of island constraints in that same language produced increased pupil dilation, indexing greater processing loads for these sentences than when hearing their grammatical counterparts. In addition, the usage-based models showed a reverse grammaticality effect with increased L2 English use, indicating diminished sensitivity to Spanish ungrammaticality in two of the three island types. This indicates that sensitivity to ungrammaticality in the L1 is attenuated by increased use of the L2, even when the ungrammaticality holds in both languages, as it does in the three island types we investigated, further suggesting that ungrammaticality in the L2 does not reinforce ungrammaticality of equivalent structures in the L1. In general, our findings align with the results reported in other studies investigating the effect of use on neurolinguistic, psycholinguistic and behavioral measures. Importantly, the effect of current use was found across participants, regardless of group adherence, while the group-based models revealed inconsistent and sometimes incoherent results, as in the case of Wh- and TA-island violations for which group analyses failed to reveal sensitivity to L1 ungrammaticality. While we do not discount group analyses as a valid method, we note that the group results obtained here may have masked the sensitivity to L1 ungrammaticality in the heritage speaker participants. Our results support the growing concern in the field that group analyses should not be the only way of investigating language processing across the speaker spectrum, i.e., for monolinguals (Tanner and Van Hell, 2014), bilinguals (Bice and Kroll, 2021) and L2 learners (Grey, 2022). Finally, we take our results to support the idea that the characterization of HS as cognitively distinct from other bilinguals is unwarranted, at least in terms of L1 processing. Usage factors have increasingly shown themselves to be significant in studies of language and should be added to other individual-level characteristics, such as relative proficiency and dominance that are likely to affect all bilingual speakers in the same way.

## Data availability statement

The raw data supporting the conclusions of this article will be made available by the authors, without undue reservation.

## Ethics statement

The studies involving human participants were reviewed and approved by IRB Graduate Center, CUNY. The patients/participants provided their written informed consent to participate in this study.

## Author contributions

GM, MJ, and CL contributed to the conception and design of the study. MJ and CL organized the database. MJ performed the statistical analysis. GM wrote the first draft of the manuscript. MJ, PF, DC, IP, and CL wrote sections of the manuscript. All authors contributed to the article and approved the submitted version.

## Funding

This work was partially funded by the New York State Education Department (NYSED) Grant #016-042, to Martohardjono.

## Conflict of interest

The authors declare that the research was conducted in the absence of any commercial or financial relationships that could be construed as a potential conflict of interest.

## Publisher’s note

All claims expressed in this article are solely those of the authors and do not necessarily represent those of their affiliated organizations, or those of the publisher, the editors and the reviewers. Any product that may be evaluated in this article, or claim that may be made by its manufacturer, is not guaranteed or endorsed by the publisher.

## Footnotes

^1^We use the term “first-acquired language” to mean the language first acquired in the home, consistently used by caregivers and in the community in which the family resides. We intend to distinguish it from the more commonly used L1 because of the connotations that this latter term has with monolingual child language and with the much-contested notion of “native speaker.” Nonetheless, for efficiency’s sake and at the suggestion of a reviewer, we use the abbreviation L1 to denote this first-acquired language.

^2^We use this term as defined in Hamers et al. (2009), to mean the ability of an individual to speak two languages, as distinct from bilingualism, which refers to the effect of two languages in contact on society as a whole.

^3^In early work, Chang et al. (2011) found that HS were better at maintaining language-internal and cross-linguistic contrasts than homeland native speakers and late learners.

^4^Note that Kałamała et al. (2022) refer to language use also as language exposure.

^5^Following linguistic convention, brackets () around material indicate optionality of that material; (*) indicates ungrammaticality of the material and *() indicates obligatory inclusion of the material.

^6^Note that this does not include a reanalysis of data from the acceptability judgment task.

^7^Complex noun phrases were not included based on syntactic literature showing indeterminacy of judgments of these structures in native speakers.

^8^For example, after hearing the item *El niño comió el dulce mientras que su tía buscaba la comida* the statement *La tia comió el dulce* appeared on the screen, followed by a Verdadero (in green) and a Falso (in red) button.

^9^While this amount of excluded data may seem high compared to behavioral methods, where more than 10% of data excluded would be rare, this is not the case for pupillometry data. For a discussion, see Schmidtke (2018: 542–543).

^10^We are of course not addressing group categorization along social parameters which may very well be applicable to the bilingual labeled “heritage speaker” by virtue of being the child of immigrants whose home language is minoritized. Minoritization of one’s first-acquired language will certainly have various implications, but of a social nature.
