# Supplementary material for: Use of the first-acquired language modulates pupil size in the processing of island constraint violations
Source: Front Psychol. 2023 Jul 14;14:1180989. doi: 10.3389/fpsyg.2023.1180989 (PMC10382202; doi:10.3389/fpsyg.2023.1180989)
Supplement: Supplementary file 1 [file Table_1.DOCX]

Supplementary Material

Use of the first-learned language modulates pupil size in the processing of island constraint violations

Gita Martohardjono*, Michael A. Johns, Pamela Franciotti, Daniela Castillo, Ilaria Porru, Cass Lowry

*** Correspondence:** Gita Martohardjono, [gmartohardjono@gc.cuny.edu](mailto:gmartohardjono@gc.cuny.edu)

**1 Language Background Questionnaire**

*Screening questions:*

1. Where were you born?
2. When did you arrive in the US?
3. How old are you?
4. What is the highest level of formal schooling you have completed?
5. Do you live alone, or with a partner?
6. Where was your partner born?
7. When did your partner arrive in the US?
8. How old is your partner?
9. What is the highest level of formal schooling your partner has completed?
10. What language do you speak at home?
11. Who were your primary caregiver(s) from birth to age 10?
12. What country were your primary caregiver(s) born in?
13. How old were your primary caregiver(s) when they arrived in the US?
14. What year did your primary caregiver(s) arrive in the US?
15. What language did you speak with your primary caregiver(s) from birth to age 10?
16. How well do you understand Spanish:

1 = little to nothing of what I hear

2 = some of what I hear

3 = about half of what I hear

4 = most of what I hear

5 = everything I hear

*Administered before first experimental session:*

1. What do you consider to be your native language?
2. Please list all the languages that you speak (DO NOT include languages that you can read but do not speak):

*For level:* 1 = I have limited knowledge of the language

2 = I have some ability to use the language

3 = I have good ability to use the language*

4 = I am a fluent speaker/user of the language

5 = I am a native speaker/user of the language

**If you select “3 = I have good ability to use the language”, please write “YES” if you are able to give an opinion and defend it in that language.*

Language ___, level 1 2 3 4 5 , when did you start learning? _ years old

Language ___, level 1 2 3 4 5 , when did you start learning? _ years old

Language ___, level 1 2 3 4 5 , when did you start learning? _ years old

Language ___, level 1 2 3 4 5 , when did you start learning? _ years old

1. For each of the above-listed languages, please describe where and how you learned it:

*Example: Language _French_: was taught in school from 1st-5th grade*

*Language __Guarani__: picked it up from friends*

Language _______________: ___________________________________

Language _______________: ___________________________________

Language _______________: ___________________________________

Language _______________: ___________________________________

1. What was the first language you learned?

_____________________

1. What languages were spoken in your house growing up?

_____________________

1. Which of the languages from (14.) were used most often?

_____________________

1. Who spoke each of the languages in (14.) to each other in your house growing up?

*Example: Language _Spanish_: everyone spoke Spanish to each other*

*Language __Nahuatl_: grandparents spoke Nahuatl to each other and no one else*

Language _______________: ___________________________________

Language _______________: ___________________________________

Language _______________: ___________________________________

Language _______________: ___________________________________

1. Please complete the following table:

| Age | What country did you live in? | What was the primary language spoken in your local community? | Did you attend school? | What was the language of instruction? |
| --- | --- | --- | --- | --- |
| 5-6 |  |  |  |  |
| 6-7 |  |  |  |  |
| 7-8 |  |  |  |  |
| 8-9 |  |  |  |  |
| 9-10 |  |  |  |  |
| 10-11 |  |  |  |  |
| 11-12 |  |  |  |  |
| 12-13 |  |  |  |  |
| 13-14 |  |  |  |  |
| 14-15 |  |  |  |  |
| 15-16 |  |  |  |  |
| 16-17 |  |  |  |  |
| 17-18 |  |  |  |  |

*Administered after second experimental session:*

1. Participant’s sex: _______________
2. Participant’s profession in U.S.: _________________________
3. Participant’s social class (choose one):

working ____ middle ____ upper____

1. Which languages do you read/write? At what level? When did you start?

*For level:* 1 = I have limited reading/writing ability in the language

2 = I have some ability to read/write in the language

3 = I have good ability to read/write in the language*

4 = I am a fluent reader/writer of the language

5 = I am a native reader/writer of the language

**If you select “3 = I have good ability to read/write in the language”, please write “YES” if you are able to defend an opinion in writing in that language.*

Language ___, level 1 2 3 4 5 , when did you start learning? _ years old

Language ___, level 1 2 3 4 5 , when did you start learning? _ years old

Language ___, level 1 2 3 4 5 , when did you start learning? _ years old

Language ___, level 1 2 3 4 5 , when did you start learning? _ years old

1. Which language(s) do you use to speak with your:
2. **father**

English / Spanish / both / N/A

1. **mother**

English / Spanish / both / N/A

1. **sisters/brothers**

English / Spanish / both / N/A

1. **children (older)**

English / Spanish / both / N/A

1. **children (younger)**

English / Spanish / both / N/A

1. **friends**

English / Spanish / both / N/A

1. **boss**

English / Spanish / both / N/A

1. **co-workers**

English / Spanish / both / N/A

1. **classmates**

English / Spanish / both / N/A

1. **significant other**

English / Spanish / both / N/A

1. How much Spanish do you use in/at:
2. **home**

mostly / little / none / N/A

1. **school**

mostly / little / none / N/A

1. **work**

mostly / little / none / N/A

1. **social activities**

mostly / little / none / N/A

1. **reading**

mostly / little / none / N/A

1. **listening to the radio/music**

mostly / little / none / N/A

1. **watching TV**

mostly / little / none / N/A

1. In a typical day, how much do you interact with the following

(please give answers as relative percentages, e.g. Spanish-speakers 75%, English-speakers 25%):

Spanish-speakers ____________ English-speakers ____________

1. Where do the interactions in (24.) occur?

Spanish-speakers: _______________________________________

English-speakers: _______________________________________

1. How often do you travel to Spanish-speaking countries?_____________
2. How long is/are your typical stay(s) in (26.) _______________
3. Do you plan on living in a Spanish-speaking country?_______________
4. Which language do you prefer (choose one):

English ____ Spanish ____ no preference ____

1. What is/are the reason(s) for your preference in (29.)?

________________________________________________________

_______________________________________________________
