# Supplementary material for: Use of the first-acquired language modulates pupil size in the processing of island constraint violations
Source: Front Psychol. 2023 Jul 14;14:1180989. doi: 10.3389/fpsyg.2023.1180989 (PMC10382202; doi:10.3389/fpsyg.2023.1180989)
Supplement: Supplementary file 2 [file Table_2.DOCX]

**Supplemental Materials: Tables**

***Wh-Islands: Group by Grammaticality***

| **Reference: HS, Grammatical** | | | | | |
| --- | --- | --- | --- | --- | --- |
| **Parametric Coefficients** | **β** | **SE** | ***t*** | ***p*** |  |
| (Intercept) | 0.00 | 0.00 | -0.67 | 0.50 |  |
|  |  |  |  |  |  |
| **Smooth Terms** | **EDF** | **Ref.DF** | ***F*** | ***p*** |  |
| s(Sample) | 3.94 | 4.66 | 4.26 | < .001 | * |
| s(Sample): IsUngram | 2.01 | 2.01 | 14.03 | < .001 | * |
| s(Sample): IsLB | 2.04 | 2.06 | 0.06 | 0.97 |  |
| s(Sample): IsUngramLB | 3.08 | 3.62 | 1.62 | 0.15 |  |
| s(X Gaze, Y Gaze) | 37.77 | 38.91 | 135.58 | < .001 | * |
| s(Sample, Subject) | 212.88 | 459.00 | 1.65 | < .001 | * |
| s(Sample, Item) | 49.02 | 300.00 | 0.65 | < .001 | * |
|  |  |  |  |  |  |
| **Reference: LB, Ungrammatical** | | | | | |
| **Parametric Coefficients** | **β** | **SE** | ***t*** | ***p*** |  |
| (Intercept) | -0.01 | 0.00 | -3.33 | 0.00 | * |
|  |  |  |  |  |  |
| **Smooth Terms** | **EDF** | **Ref.DF** | ***F*** | ***p*** |  |
| s(Sample) | 3.94 | 4.65 | 4.78 | < .001 | * |
| s(Sample): IsGram | 2.00 | 2.01 | 1.57 | 0.19 |  |
| s(Sample): IsHS | 2.64 | 2.86 | 0.66 | 0.58 |  |
| s(Sample): IsGramHS | 2.01 | 2.01 | 2.10 | 0.11 |  |
| x(X Gaze, Y Gaze) | 37.77 | 38.91 | 135.55 | < .001 | * |
| s(Sample, Subject) | 212.84 | 458.00 | 1.66 | < .001 | * |
| s(Sample, Item) | 48.76 | 299.00 | 0.65 | < .001 | * |

***Temporal Adverbial Islands: Group by Grammaticality***

| **Reference: HS, Grammatical** | | | | | |
| --- | --- | --- | --- | --- | --- |
| **Parametric Coefficients** | **β** | **SE** | ***t*** | ***p*** |  |
| (Intercept) | -0.02 | 0.00 | -4.69 | 0.00 | * |
|  |  |  |  |  |  |
| **Smooth Terms** | **EDF** | **Ref.DF** | ***F*** | ***p*** |  |
| s(Sample) | 3.45 | 4.05 | 3.16 | 0.01 | * |
| s(Sample): IsUngram | 4.82 | 5.82 | 0.87 | 0.42 |  |
| s(Sample): IsLB | 2.01 | 2.01 | 6.94 | < .001 | * |
| s(Sample): IsUngramLB | 2.07 | 2.12 | 13.25 | < .001 | * |
| s(X Gaze, Y Gaze) | 38.57 | 38.99 | 307.78 | < .001 | * |
| s(Sample, Subject) | 208.67 | 468.00 | 1.72 | < .001 | * |
| s(Sample, Item) | 105.10 | 299.00 | 0.96 | < .001 | * |
|  |  |  |  |  |  |
| **Reference: LB, Ungrammatical** | | | | | |
| **Parametric Coefficients** | **β** | **SE** | ***t*** | ***p*** |  |
| (Intercept) | -0.02 | 0.00 | -4.90 | 0.00 | * |
|  |  |  |  |  |  |
| **Smooth Terms** | **EDF** | **Ref.DF** | ***F*** | ***p*** |  |
| s(Sample) | 3.04 | 3.60 | 3.32 | 0.02 | * |
| s(Sample): IsGram | 6.30 | 7.46 | 8.63 | < .001 | * |
| s(Sample): IsHS | 2.01 | 2.01 | 0.09 | 0.91 |  |
| s(Sample): IsGramHS | 2.70 | 3.09 | 10.60 | < .001 | * |
| s(X Gaze, Y Gaze) | 38.57 | 38.99 | 307.55 | < .001 | * |
| s(Sample, Subject) | 207.84 | 469.00 | 1.71 | < .001 | * |
| s(Sample, Item) | 104.42 | 300.00 | 0.95 | < .001 | * |

***Relative Clause Islands: Group by Grammaticality***

| **Reference: HS, Grammatical** | | | | | |
| --- | --- | --- | --- | --- | --- |
| **Parametric Coefficients** | **β** | **SE** | ***t*** | ***p*** |  |
| (Intercept) | -0.02 | 0.00 | -5.98 | < .001 | * |
|  |  |  |  |  |  |
| **Smooth Terms** | **EDF** | **Ref.DF** | ***F*** | ***p*** |  |
| s(Sample) | 3.87 | 4.50 | 5.71 | < .001 | * |
| s(Sample): IsUngram | 2.01 | 2.01 | 9.16 | < .001 | * |
| s(Sample): IsLB | 2.01 | 2.02 | 2.20 | 0.11 |  |
| s(Sample): IsUngramLB | 2.01 | 2.02 | 3.94 | 0.02 | * |
| s(X Gaze, Y Gaze) | 38.49 | 38.98 | 499.81 | < .001 | * |
| s(Sample, Subject) | 238.48 | 510.00 | 2.22 | < .001 | * |
| s(Sample, Item) | 169.52 | 450.00 | 1.08 | < .001 | * |
|  |  |  |  |  |  |
| **Reference: LB, Ungrammatical** | | | | | |
| **Parametric Coefficients** | **β** | **SE** | ***t*** | ***p*** |  |
| (Intercept) | -0.02 | 0.00 | -4.14 | < .001 | * |
|  |  |  |  |  |  |
| **Smooth Terms** | **EDF** | **Ref.DF** | ***F*** | ***p*** |  |
| s(Sample) | 3.87 | 4.49 | 6.86 | < .001 | * |
| s(Sample): IsGram | 2.01 | 2.02 | 26.82 | < .001 | * |
| s(Sample): IsHS | 2.01 | 2.02 | 0.09 | 0.92 |  |
| s(Sample): IsGramHS | 2.02 | 2.03 | 3.88 | 0.02 | * |
| s(X Gaze, Y Gaze) | 38.51 | 38.98 | 563.63 | < .001 | * |
| s(Sample, Subject) | 238.66 | 510.00 | 2.23 | < .001 | * |
| s(Sample, Item) | 169.57 | 450.00 | 1.08 | < .001 | * |

***Wh-Islands: Usage by Grammaticality***

The model summary below represents the model for usage by grammaticality for the Wh-islands, but with the non-significant interaction with historical usage removed.

| **Current Usage by Grammaticality (No Historical Usage)** | | | | | |
| --- | --- | --- | --- | --- | --- |
| **Parametric Coefficients** | **β** | **SE** | ***t*** | ***p*** |  |
| (Intercept) | 0.00 | 0.00 | 0.07 | 0.95 |  |
|  |  |  |  |  |  |
| **Smooth Terms** | **EDF** | **Ref.DF** | ***F*** | ***p*** |  |
| s(Sample) | 3.96 | 4.72 | 5.89 | 0.00 | * |
| s(Sample): IsUngram | 1.01 | 1.01 | 0.09 | 0.77 |  |
| s(Historical Usage) | 1.00 | 1.00 | 1.09 | 0.30 |  |
| s(Historical Usage): IsUngram | 2.01 | 2.01 | 1.53 | 0.22 |  |
| s(Current Usage) | 1.41 | 1.46 | 2.13 | 0.09 |  |
| s(Current Usage): IsUngram | 2.79 | 3.27 | 9.01 | < .001 | * |
| ti(Sample, Current Usage) | 2.15 | 2.32 | 3.76 | 0.02 | * |
| ti(Sample, Current Usage): IsUngram | 4.86 | 6.51 | 4.47 | < .001 | * |
| s(X Gaze, Y Gaze) | 37.71 | 38.90 | 120.42 | < .001 | * |
| s(Sample, Subject) | 157.77 | 388.00 | 1.42 | < .001 | * |
| s(Sample, Item) | 62.76 | 300.00 | 0.37 | < .001 | * |
